# Neural and behavioral alterations of a real-time interpersonal distance (IPD) development process in differing social status interactions

**DOI:** 10.3389/fnbeh.2022.969440

**Published:** 2022-10-13

**Authors:** Xinxin Huang, Shin-Ichi Izumi, Yoshimi Suzukamo

**Affiliations:** ^1^Department of Physical Medicine and Rehabilitation, Tohoku University Graduate School of Medicine, Sendai, Japan; ^2^Department of Physical Medicine and Rehabilitation, Graduate School of Biomedical Engineering, Tohoku University, Sendai, Japan

**Keywords:** social status, real-time, interpersonal distance (IPD), development process, fNIRS, teacher-student

## Abstract

**Background:**

Evidence showed neural changes in interpersonal distance (IPD) interaction, and neural activities are affected by relationships (such as friends or strangers). Behavior studies proved that social status strongly affects IPD between two persons. However, how the differing social status impacts neural alterations in the IPD interactions remains unknown.

**Objectives:**

The teacher-student relationship is a typical representation of the difference in social status. The present study aims to investigate the IPD performance and brain processes underlying real-time differing social status during the development process from teacher-student interactions.

**Materials and methods:**

We designed three within-subject experiments corresponding to the inclusion, control, and affection stages of IPD. Altogether, 38 valid healthy participants participated in three experiments with a teacher (differing social status condition, DS condition) and a peer student (peer social status condition, PS condition) separately. This study employed functional near-infrared spectroscopy (fNIRS) and modified real-time stop-distance paradigms to record IPD performance and neural processes.

**Results:**

For IPD performance, significantly larger IPD gaps were shown in the DS condition than in the PS condition, and IPD feedback affected IPD performance. For neural alterations, activated frontopolar area (FPA, BA10), dorsolateral prefrontal cortex (DLPFC, BA9/BA46), and Broca’s area (BA45) were observed across the IPD stages. Importantly, brain activation shifts with the development of IPD. In addition, results showed that differences in Oxy-Hb changes were located in the FPA (BA10), DLPFC (BA9/BA46), and Broca’s area (BA45) between the DS and PS conditions across IPD stages. Additionally, negative correlations were found between Oxy-Hb changes and IPD performance.

**Conclusion:**

We propose prefrontal cortex (PFC) and Broca’s area involvement in IPD interactions, initially focusing on evaluation and action periods, and later on IPD-evaluation processes after feedback. In addition, a difference in Oxy-Hb activities implies the complexity of relationships and social status in IPD interactions.

## Introduction

Interpersonal distance (IPD) is the physical distance between individuals during which rich and variable non-verbal social interactions occur. IPD provides spatial information visible during real-time interpersonal interactions (Kroczek et al., 2020). IPD interactions reflect the nature of the relationship in a non-verbal way, providing a vivid and straightforward perspective to understand social interactions. IPD interactions are prevalent in social relationships, such as mother-infant (Guida et al., 2021), friends-strangers (Haim-Nachum et al., 2021), and lovers (Akbarian et al., 2020). In the complex IPD interaction, differing social status IPD interaction is a significant and essential social interaction and plays a crucial role in personal development. A comprehensive and systematic comprehension of the neural basis for differing social status IPD interaction contributes to enriching the understanding of social cognition and making sense to construct harmonious social life. However, little is known about the nature of differing social status IPD interactions in real-time contexts, especially regarding the neural basis.

In the last decade, cognitive neuroscience has proven that the prefrontal cortex (PFC) is a biomarker for differing social status social interactions. Numerous studies have examined PFC activities during differing social status social interaction, such as trust games (Cheng et al., 2022) and problem-solving tasks (Takeuchi et al., 2019) using functional near-infrared spectroscopy (fNIRS). Furthermore, a fMRI study showed medial prefrontal cortex (MPFC) activity in differing social status social feedback interactions (Nakagawa et al., 2021). In addition, neural changes in the PFC of status gap pairs were significantly greater than those in non-status gap pairs during a cooperation drawing task (Sun et al., 2021). The mentioned studies highlighted the PFC as a biomarker during differing social status interactions from verbal to non-verbal communication. Non-verbal communication studies are crucial for understanding human behavior. Non-verbal behaviors include eye contact, gestures, facial expressions, and IPD. The IPD interaction plays a vital role in making comprehension of interpersonal interactions. However, little is known about the underlying neural activities in real-time IPD interactions between differing social status during the development process.

Neural research on IPD interactions has consistently revealed brain activity in the parietal lobe, motor areas, PFC, and amygdala (Huang and Izumi, 2021), although different IPD tasks have been adopted. The PFC has been implicated in IPD behavior, which is essential for interpersonal interactions. For example, a recent study indicated increased PFC activity between friends’ and strangers’ IPD approach (Cohen et al., 2018). Moreover, other studies have shown an enhanced DLPFC in facial approach-approached stimuli (Schienle et al., 2017). Besides, evidence proved that PFC activities under passive IPD interactions through fMRI and electroencephalogram (EEG) (Huang and Izumi, 2021) but did not explore the PFC changes under real-time IPD interactions due to the limitation of the methods. A method such as fNIRS is required to record neural activity during real-time ecological IPD interactions. Compared to fMRI and EEG, fNIRS has the advantage of anti-motion artifacts and is suitable for natural experimental applications (Cui et al., 2011). fNIRS measures oxygenated hemoglobin (Oxy-Hb) changes in naturalistic interactions such as conversation (Takei et al., 2014) and non-verbal communication (Noah et al., 2017). The present study explores Oxy-Hb changes by fNIRS to understand the neural basis of differing social status IPD interactions. Besides, the IPD interaction is a complex social process, including cognitive evaluation, IPD action, or evaluation and adjustment. Passive IPD paradigms cannot reflect those perspectives. To rich the nature of IPD interaction, the present study investigates the PFC changes in evaluation and action process under a variety of IPD interactions.

Additionally, little is known about differing social status IPD interactions from a relationship development perspective. IPD is a reflection and derived external manifestation of interpersonal relationships. As relationships develop and change, IPD changes along with them. To understand the development process of IPD, the current study draws on a classical, and famous interpersonal development theory called fundamental interpersonal relations orientation (FIRO), which was introduced by Schutz (1958). The FIRO theory posits that the development of a relationship occurs in three stages based on relationship needs: inclusion, control, and affection. The initial stage is inclusion, which means that an individual wants to belong to or be included by others. Next is the control stage, which is when the individual wants to control or be controlled by others in the interactions. The last stage is the affection stage, during which individuals send likeness or love to others or want to be liked by others. These three processes illustrate the development of interpersonal relationships, and we designed three experiments that correspond to the three stages of IPD development. Exploring differing social status IPD based on the FIRO theory makes sense for a systematic comprehension of differing social status IPD interactions.

The present study adopted the fNIRS technique and ecological IPD interaction paradigms to investigate the behavior and PFC changes of differing social status IPD interactions during the development process. In the current study, the differing social status was from the social status gaps between teacher and student as this kind of social relationship represents differing social status typically. We tested a series of hypotheses across three IPD stages. Above all, we expected the IPD performance in the differing social status condition (DS condition) would be larger than the peer social status condition (PS condition). And the IPDs would narrow along with the IPD stages. If we observed a smaller IPD in the IPD-Affection stage compared to IPD-Inclusion stage, it means the manipulation of the IPD-development was successful from the behavioral perspective. Moreover, we hypothesized the IPD feedback from others impacts the IPD performance. Additionally, previous studies have examined PFC activity in differing social status interactions (Cheng et al., 2022) and IPD cognition (Vieira et al., 2017). The present study set the PFC as the region of interest (ROI) to observe the changes in the PFC. We expected to find PFC activations during the IPD interaction across three IPD stages under, and the PFC activations would change along with the IPD stages. In addition, as the teacher stands for the authority and power, we hypothesized that greater neural changes were seen in the DS condition than in the PS condition in the three IPD stages. As for student participants, the IPD interactions with teachers would be a heavy and complex cognition task. Lastly, we examined the correlations between IPD performance and neural changes. IPD-neural studies showed the inconsistent results, and the strength of the correlation was medium. In the present real-time study, we record the neural changes along with the real-time IPD interaction, we expected a high association in the IPD performance and neural changes in the IPD interactions.

## Materials and methods

### Participants

The inclusion criteria were as follows: (1) right-handed, (2) student identity, (3) physically and psycho-healthy individuals, and (4) Asian students. Students (1) with a formal job or job experience, (2) who had a history of psychiatric or neurological disorders, (3) who were receiving psychotherapy and medical treatment, or (4) who came from Western countries were excluded.

We conducted a prior power analysis to confirm the sample size using G*Power 3.1 (Faul et al., 2007). For an α of 0.05 and a power (1-β) of 0.90, it was determined that 36 participants would detect an interaction of moderate effect size (η^2^ = 0.25). In case the participants did not meet the analysis requirements, we recruited 40 participants. Forty healthy participants from Tohoku University were enrolled through advertisements, but two did not finish all the experiments, leaving 38 participants. The respondents received a 3000 YEN gift card. Nineteen men (Age: 24.4 ± 1.1 years, Height: 173.3 ± 5.2 cm) and nineteen women (Age: 24.2 ± 1.6 years, Height: 165.6 ± 5.9 cm), comprising seven undergraduates and thirty-one graduates, participated in the study. The participants were Chinese, Japanese, and Malaysian people.

Participants were randomly assigned to four pairing contexts based on enrollment in order to eliminate gender effects. Ten participants were paired with the male teacher (63 years old and 172 cm) and the male student (23 years old and 173 cm); nine participants were paired with the male teacher and the female student (24 years old and 163 cm); ten participants were paired with the female teacher (60 years old and 162 cm with a high heel) and the male student; and nine participants were paired with the female teacher and the female student. The teachers and students were real teachers and peer students who participated in the experiments as supporters. To distinguish identities, teachers wear business shirts and black trousers, and students wear casual clothes (white tops and black trousers) throughout all the experiments. All participants were meeting the teachers for the first time; two participants had met the male student and two participants had met the female student once before, but they were unfamiliar acquaintances. The ethical committee of Tohoku University (Sendai, Japan) approved this research, and participants provided written informed consent to participate in the experiments.

### Measurements

#### Interpersonal distance measurements

The IPD between the participants’ feet and the teacher/peer student was measured using a digital laser ruler (RYOBI, LDM-500) and two wooden blocks (Supplementary Figure 1).

#### Questionnaire

To evaluate the most comfortable IPD in the IPD-deprivation and IPD-controllable processes, participants were asked to select answers for the following questions:

What was the most comfortable distance for you when you just stood up and were approached by the teacher/student? A. 1.2; B. 0.6; and C. 0.3 m.

What was the most comfortable distance for you when you approached the teacher/student? A. 1.2; B. 0.6; and C. 0.3 m.

#### Functional near-infrared spectroscopy measurements

**FIGURE 1:**
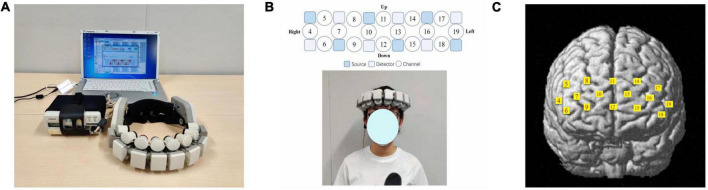
The setting of the NIRS device. **(A)** The WOT-100 device, including the handset, mobile control box, net-box, and measurement controller. **(B)** An example of a participant wearing the WOT-100 device on the frontal head, and the source, detector, and channel setting of the WOT-100. **(C)** The spatial registration of each channel on the frontal cortex.

Wearable 16-channel NIRS equipment (WOT-100, Hitachi Company, Tokyo, Japan) was used to evaluate the concentrations of Oxy-Hb and deoxygenated hemoglobin (Deoxy-Hb) in the PFC, adopting wavelengths of near-infrared light (705 and 830 nm). The sampling rate was set at 5 Hz. A flexible cable bundle was connected, and a probe unit was used to record the optical topography. A personal computer received data from the portable processing unit through a wireless local area network (Figure 1A). The NIRS probes and channel settings are shown in Figure 1B. The WOT-100 NIRS device consisted of six emitters and six detectors, contributing to 16 channels, each channel with one source–detector pair. The distance between the source and detector probes of each channel was set at 3.0 cm. Based on the international 10–20 system of electroencephalography, the lowest probe was positioned along the Fp1–Fp2 line. A previous study used a three-dimensional digitizer to validate channel positions using the Montreal Neurological Institute coordinate system (Funane et al., 2011). Each channel was then registered into the standard brain template according to the NIRS-SPM toolbox based on SPM 8 and MATLAB 13.0 (Korea Advanced Institute of Science and Technology, Korea). Detailed MNI positions are shown in Figure 1C and Supplementary Table 1.

### Procedures

**FIGURE 2:**
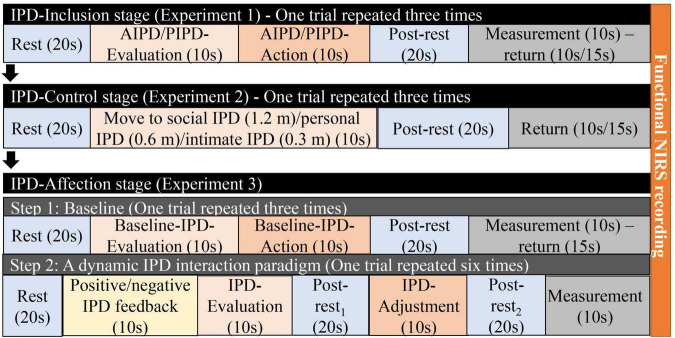
Experiment procedures for differing social status IPD interactions. Procedures from experiment 1, experiment 2, and experiment 3.

The IPD interaction paradigms were designed using the stop-distance paradigm (Hayduk, 1978), a well-validated measurement of IPD. Each participant started from the IPD-Inclusion stage (Experiment 1), then the IPD-Control stage (Experiment 2), and ended with the IPD-Affection stage (Experiment 3) in sequence to experience the development of the IPD progress with a teacher and peer student separately (Figure 2). The order of pairing with a teacher or peer student was counterbalanced. The study was conducted in a quiet and professional lab of Tohoku University. Before the experiment started, the participant and teacher/peer student had a simple greeting with each other and knew names and identities under the introduction of the experimenter. In the DS condition, the experimenter introduced the teacher to the participant: “This is teacher A; he/she will finish all the experiments with you.” In the PS condition, the experimenter introduced the peer student to the participant: “This is B; he/she will finish all the experiments with you.” The participants wore the WOT-100 NIRS equipment to record brain activity throughout the experimental processes. Before each experiment, the experimenter explained the experiment and the instructions until the participants fully understood. We recorded the experimental instructions and played the corresponding recording for each experiment, and the participants performed the tasks following the recorded instructions. In the three experiments, the initial IPD between participants and teachers/students was three meters, the participants and teachers/students did not make eye contact, and the teacher/student looked at the knee of the participant, maintaining a neutral expression (Supplementary Figure 2).

**FIGURE 3:**
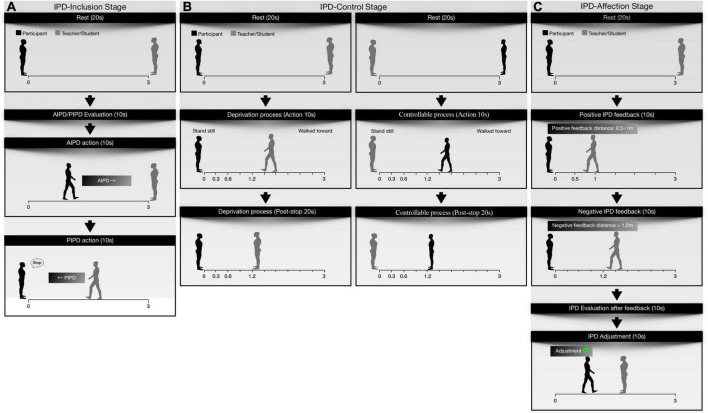
Scenes of core experimental procedures. **(A)** Active interpersonal distance (AIPD) processes and passive interpersonal distance (PIPD) processes in experiment 1. **(B)** Before and after social IPD (1.2 m) procedure in experiment 2 in both deprivation and control processes. **(C)** Positive IPD feedback and negative IPD feedback from the partner and the IPD action (after feedback) of the participant in experiment 3.

Interpersonal distance-inclusion stage: this stage contained active interpersonal distance (AIPD) and passive interpersonal distance (PIPD) (Figure 3A). In the AIPD task, the participants had 10 s to mentally evaluate the most comfortable distance between themselves and the teacher/peer students (active interpersonal distance evaluation period, AIPD-E). The participant then approached the teacher/student and stopped at the most comfortable distance (active interpersonal distance action period, AIPD-A). AIPD was measured with a laser ruler. In the PIPD task, the participant had 10 s to evaluate the most comfortable distance when approached by the teacher/student (passive interpersonal distance evaluation period, PIPD-E). The participant was then approached by the teacher or student at a slow speed. Once the teacher/student arrived at the most comfortable distance for the participant, the participant said “stop,” and the teacher/student stopped (passive interpersonal distance action period, PIPD-A). The experimenter recorded the PIPD. The trial was repeated thrice.

Interpersonal distance-control stage: after resting for 5 min, the participants continued the IPD-Control stage (Figure 3B). First, participants joined an IPD-deprivation process, during which they stood still. The teacher/student walked slowly toward the participant for 10 s (action period) and then stopped at the social IPD (1.2 m) for 20 s (post-stop period). The teacher/student then returned to the starting line and restarted walking to and stopping at a social IPD (1.2 m), personal IPD (0.6 m), and intimate IPD (0.3 m), respectively. The IPD deprivation trial was repeated thrice. After resting for 5 min, the participants participated in the IPD-controllable processes. The roles of the teacher and the student were exchanged. Participants took 10 s to walk slowly toward the teachers/students and stopped at social IPD (1.2 m)/personal IPD (0.6 m)/intimate IPD (0.3 m) for 20 s. The IPD-controllable trial was repeated thrice. The IPD was pre-designed at 1.2, 0.6, and 0.3 m, corresponding to social distance (1.2–3.7 m), personal distance (0.46–1.2 m), and intimate distance (0–0.46 m) from Hall’s social distance theory (Hall, 1963). After this stage, participants completed a questionnaire.

Interpersonal distance-affection stage: after resting for 10 min, the participants continued the IPD-Affection stage (Figure 3C). (1) The baseline measurement was the same as that of the AIPD task in the IPD-Inclusion stage containing the baseline-IPD evaluation and baseline-IPD action periods. Participants were instructed that they had 10 s to evaluate the most comfortable distance between them and their partner in their mind (Baseline-IPD evaluation). Participants then walked toward their partner at a slow speed and stopped at the most comfortable distance between them and their partner according to their feelings (Baseline-IPD action). The baseline trial was repeated thrice. (2) In the dynamic IPD interaction paradigm, participants were told to take a turn to adjust to the most comfortable distance, and each person had six opportunities in total. At each opportunity, after the movement of the teacher/student, the participants could choose to step forward, back, or stand still to adjust the IPD between them and their partner. The teacher or student started the action. Then, participants had 10 s to evaluate and act to the IPD to their comfortable IPD (evaluation period after feedback) before adjusting the IPD in another 10 s (action period after feedback). Each IPD feedback action of the teacher or student was predesigned. The IPD feedback action from teachers/students was divided into positive and negative IPD feedback actions. In the positive IPD feedback action, participants were approached by the teacher/student with an IPD between 0.5 and 1 m. In the negative IPD feedback action, participants were approached by the teacher/student with an IPD larger than 1.2 m. The design of the two kinds of IPD feedback was based on Hall’s social distance theory (Hall, 1963) and was combined with laboratory size. Positive IPD feedback and negative IPD feedback each appeared three times in total. The sequence in which the presentation of the two types of IPD feedback occurred was randomized. The experimenter recorded the IPD using a laser ruler every time the IPD was changed.

### Data analysis

#### Near-infrared spectroscopy data processing

Oxygenated hemoglobin was chosen as the indicator of NIRS data because it is the most sensitive measure of changes in regional cerebral blood flow (Hoshi et al., 2001). Processing procedures: (1) Data trimming: we left rest periods, task periods, and post-rest periods but cut the measurement and return periods (Figure 2; colored blocks were included, and gray blocks were excluded). In the IPD-Inclusion stage, rest, evaluation, action, and post-rest period were left as new continuous raw data after trimming. In the IPD-Control stage, rest, IPD action period, and post-rest periods were left as new continuous raw data after trimming. In the IPD-Affection stage, rest, IPD-evaluation (baseline), IPD-action (baseline), IPD feedback, IPD-evaluation (after feedback), post-rest_1_, IPD-action (after feedback), and post-rest_2_ were left as new continuous raw data after trimming. The new continuous raw data was utilized in the next step. (2) High-pass filtering: Data were processed using Wavelet-MDL detrending (minimum description length) to remove global trends caused by breathing, heartbeat, vasomotion, and experimental errors (Jang, 2009). (3) Low-pass filtering: The hemodynamic response function (HRF) smoothed the data. (4) Individual-level analysis: General linear model (GLM) parameters and temporal correlations were estimated for the individual-level analysis. The average beta value of 16 channels was calculated based on the average of task periods. The IPD-Inclusion stage includes the AIPD evaluation periods, PIPD evaluation periods, AIPD action periods, and PIPD action periods; The IPD-Control stage includes the social IPD (1.2 m) action periods, personal IPD (0.6 m) action periods, intimate IPD (0.6 m) action periods; The IPD-Affection stage includes the IPD evaluation periods (baseline), IPD action periods (baseline), IPD-evaluation periods (after feedback), and IPD-action periods (after feedback). The beta value is the weight coefficient in the GLM and represents the hemodynamic response curve to model the Oxy-Hb changes. Beta-values were extracted for further analysis. NIRS data were obtained using the NIRS-SPM toolbox (Ye et al., 2009), SPM8, and MATLAB 2013b (MATHWORKS, Natick, MA, United States).

#### Statistical analyses

For the behavioral data analysis, Cronbach’s α and intraclass correlation coefficient (ICC) were adopted in the IPD-Inclusion stage to validate the reliability of IPD performance using the stop-distance paradigm. We then calculated the average IPD of the three trials as IPD indicators in the IPD-Inclusion stage, including the average AIPD and average PIPD. Finally, a two-way repeated-measures ANOVA [2 (social status: DS, PS) × 2 (IPDs: AIPD, PIPD)] was performed. Height and age were used as the covariates. To test the effectiveness of the experimental design for IPD development, we conducted a paired *t*-test between the AIPD in the IPD-Inclusion stage and the baseline-IPD in the IPD-Affection stage. Five participants were excluded from the IPD feedback analysis because of the failure of the experiment in the IPD-Affection stage; the participants suspected what the operation of the IPD feedback was and the wrong IPD was sent by the teacher/student. Thus, 33 participants were included in the IPD feedback effects analysis in the IPD-Affection stage. We then adopted two indicators, ΔPOS-IPD and ΔNEG-IPD, to test the IPD feedback effects between differing social status. The ΔPOS/NEG-IPD calculated as positive/negative IPD (adjusted distance after receiving positive/negative IPD feedback) minus the baseline IPD indicated the changes in IPD value after the action of positive/negative IPD feedback. Then, a two-way [social status: (DS, PS) × feedback (positive, negative)] repeated-measures ANOVA was performed.

For the questionnaire analysis, we used a chi-squared test for each condition. Three participants were excluded from the analysis because of incomplete responses.

For the neural change analysis, the beta value of Oxy-Hb changes was extracted from the processed NIRS data. A box-plot was then adopted for detecting the outliers for the beta value in each channel, and the outliers were set as missing values and replaced by the linear trend at those points. Some data were excluded due to damage or missing data and bad channel signals (Supplementary Table 2). (1) One-sample *t*-tests. Average Oxy-Hb changes of each channel in tasks were compared to the 0 value separately in the IPD-Inclusion and IPD-Control stages. Tasks in the IPD Inclusion stage, including the AIPD-E, PIPD-E, AIPD-A, and PIPD-A. Tasks in the IPD-Control stage, including the social IPD (1.2 m), personal IPD (0.6 m), and intimate IPD (0.3 m). (2) Paired *t*-tests. Average Oxy-Hb changes in the evaluation and action periods after positive/negative feedback were compared with baseline (evaluation/action) to test the activation channels after feedback in the Affection stage. Channels with positive *t*-values (*p* ≤ 0.05) were confirmed as the activated channel. The *p*-values in the *t*-tests were adjusted by the false discovery rate (≤0.05). Activation results were visualized by the xjView software^1^ and the BrainNet Viewer toolbox (Xia et al., 2013). (3) Three-way repeated-measure ANOVA. The beta value of the Oxy-Hb changes was analyzed using a three-way repeated-measure ANOVA of each channel in each IPD stage. However, in the IPD-Affection stage, [Oxy-Hb] was set as the indicator; it used the beta value of the IPD evaluation/action period after positive/negative feedback minus the beta value of the baseline (evaluation/action period). In the IPD-Inclusion stage, a three-way repeated-measures ANOVA [2 (social status: DS, PS) × 2 (IPDs: AIPD, PIPD) × 2 (periods: evaluation, action)] was done. In the IPD-Control stage, a three-way repeated-measures ANOVA {2 (social status: DS, PS) × 2 (process: deprivation, controllable) × 3 [IPDs: social IPD (1.2 m), personal IPD (0.6 m), intimate IPD (0.3 m)}] was done. In the IPD-Affection stage, a three-way repeated measure ANOVA [2 (social status: DS, PS) × 2 (feedback: positive, negative) × 2 (periods: evaluation, action)] was done. Repeated-measures ANOVA analyses were used to investigate the main effect and interaction effects, followed by multiple Bonferroni comparisons. A simple effect analysis was performed if the results showed an interaction effect.

Pearson’s correlations were computed for each channel to determine the relationship between PFC activity and IPD performance. To further investigate the data, linear regression analysis was performed to examine the effects of Oxy-Hb changes during IPD interactions.

The statistical significance level was set at a *p*-value of ≤0.05. Statistical analyses were performed before March 2022 using the SPSS statistics software 22.0 (IBM Corporation, Armonk, NY, United States).

## Results

### Interpersonal distance measurements

**FIGURE 4:**
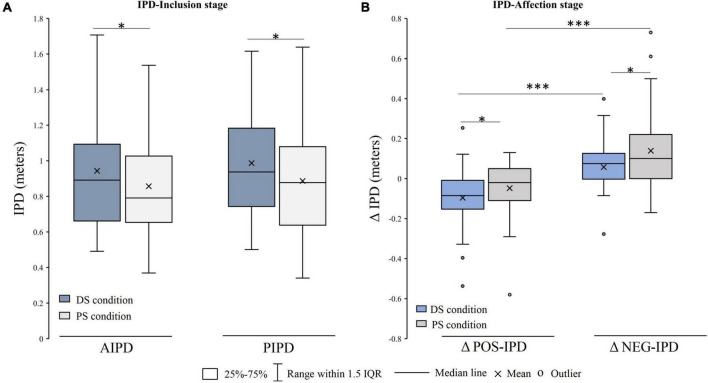
Interpersonal distance (IPD) difference between DS condition and PS condition. **(A)** IPD difference in the IPD-Inclusion stage. **(B)** ΔIPD difference in the IPD-affection stage. DS, differing social status; PS, peer social status; AIPD, active interpersonal distance; PIPD, passive interpersonal distance; ΔPOS-IPD/NEG-IPD, the IPD after positive/negative IPD feedbacks minus the baseline; IQR, interquartile range; *p < 0.05; ^***^p < 0.001.

Interpersonal distance-inclusion stage: the results of the IPD exhibited good reliability. The Cronbach’s α of the IPD ranged from 0.947 to 0.968 (*r* > 0.80) and the ICC ranged from 0.855 to 0.909 (ICC > 0.80). ANOVAs showed significant main effects of the relationship, *F* (1, 35) = 6.370, *p* = 0.016, η^2^ = 0.154. The *post-hoc* analysis showed that the IPD of the DS condition was larger than that of the PS condition (*p* = 0.041, *p* < 0.05). Moreover, the main effect of the IPD was marginally significant, *F* (1, 35) = 3.580, *p* = 0.067, and η^2^ = 0.093. No significant interaction effects were observed (Figure 4A).

Interpersonal distance-affection stage: the baseline IPD in the IPD-Affection stage (0.799 ± 0.233) was significantly shorter than the initial IPD in the IPD-Inclusion stage [0.857 ± 0.285, *t* = 2.430 (37), *p* = 0.02] in the PS condition. There was no significant difference between the initial IPD (0.944 ± 0.313) and baseline IPD [0.921 ± 0.281, *t* = 1.013 (37), *p* = 0.318] in the DS condition. The ANOVA results showed the main effects of the relationship [*F* (1, 32) = 5.517, *p* = 0.025, η^2^ = 0.147] and IPD feedback [*F* (1, 32) = 53.856, *p* < 0.001, η^2^ = 0.627]. The *post-hoc* analysis showed that the changes in IPD values (ΔPOS-IPD and ΔNEG-IPD) in the DS condition were smaller than those in the PS condition. In addition, the ΔNEG-IPD was larger than the ΔPOS-IPD for the two conditions. No interaction effects were observed (Figure 4B).

### Questionnaire measurements

The results showed a significant difference in the IPD-controllable processes of PS conditions in the IPD-Control stage (χ^2^ = 24.4, *p* < 0.001); 25 participants chose personal IPD (0.6 m) as the most comfortable IPD. No significant result was found in the teacher (deprivation/controllable) and student (deprivation) conditions (Supplementary Table 3).

### Activation channels in each interpersonal distance stage

**FIGURE 5:**
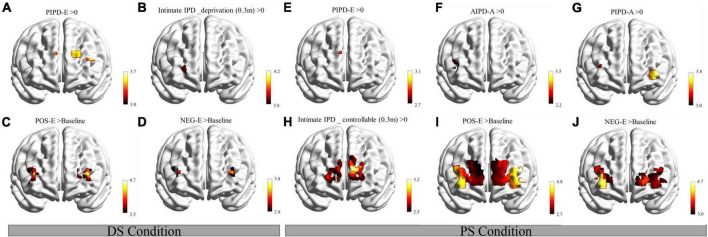
Activation maps in the three IPD stages of the DS and PS conditions. **(A)** Activation maps in the IPD-Inclusion stage of the DS condition. **(B)** Activation maps in the IPD-control stage (deprivation process) of the DS condition. **(C,D)** Activation in the IPD-Affection stage of the DS condition. **(E–G)** Activation maps in the IPD-inclusion stage of the PS condition. **(H)** Activation maps in the IPD-control stage (controllable process) of the PS condition. **(I,J)** Activation in the IPD-affection stage of the PS condition. DS, differing social status; PIPD-E, passive interpersonal distance during the evaluation period; AIPD-A, active interpersonal distance during the action period; PIPD-A, passive interpersonal distance during the action period; intimate IPD_deprivation (0.3 m), intimate IPD (0.3 m) during deprivation process; intimate IPD_controllable (0.3 m), intimate IPD (0.3 m) during controllable process; POS-E, IPD evaluation period after positive feedback; NEG-E, IPD evaluation period after negative feedback. The vertical color scale indicates the statistical significance of the *T*-values. >, compared to. Significance of all maps were set as FDR_*p* ≤ 0.05.

Interpersonal distance-inclusion stage: in the DS condition, the results revealed significant activations in channels 11 (located in the DLPFC/FPA, BA9/BA10) and 17 (located in the left Broca’s area/DLPFC, BA45/BA46) during the PIPD-E period (Figure 5A). In the PS condition, results showed activated channel 11 (BA9/BA10) during the PIPD-E period (Figure 5E); activated channel 4 (located in the right Broca’s area/DLPFC, BA45/BA46) during the AIPD-A period (Figure 5F); activated channels 7 (located in the right FPA/DLPFC, BA10/BA46) and 18 (located in the left FPA/DLPFC/inferior prefrontal gyrus, BA10/BA46/BA47) during the PIPD-A period (Figure 5G). There were no significant results found in other channels. *T*-test results are shown in Supplementary Table 4.

Interpersonal distance-control stage: in the DS condition, results showed activation results in channel 9 (located in the right FPA/Orbitofrontal area, BA10/BA11) in the intimate IPD (0.3 m) during the deprivation process (Figure 5B). In the PS condition, significant activation results were shown in channels 9 (BA10/BA11), 11 (BA9/BA10), 12 (located in the FPA, BA10), 13 (located in the FPA, BA10), and 14 (located in the left DLPFC/FPA, BA9/BA10/BA46) in the intimate IPD (0.3 m) during the controllable process (Figure 5H). There were no significant channels shown in the DS and PS conditions. *T*-test results are shown in Supplementary Table 5.

Interpersonal distance-affection stage: in the DS condition, the results revealed activations in channels 6 (located in the right FPA/DLPFC, BA10/BA46), 7 (BA10/BA46), and 16 (located in the left FPA/DLPFC, BA10/BA46) in the comparison of Oxy-Hb changes after positive feedback and baseline during the evaluation period (Figure 5C); results also showed significant channels 4 (BA45/BA46), 7 (BA10/BA46), and 16 (BA10/BA46) in Oxy-Hb changes after negative feedback compared to baseline during the evaluation period (Figure 5D). In the PS condition, activated channels 4 (BA45/BA46), 5 (located in the right Broca’s area/DLPFC, BA45/BA46), 6 (BA10/BA46), 7 (BA10/BA46), 14 (BA9/BA10/BA46), 15 (located in the left FPA/Orbitofrontal area, BA10/BA11), 16 (BA10/BA46), 17 (BA45/BA46), 18 (BA10/BA46/BA47), and 19 (located in the left Broca’s area/DLPFC, BA45/BA46) were shown in the paired *t*-test of Oxy-Hb changes after positive feedback and baseline during the evaluation period (Figure 5I); results also revealed significant results in channels 4 (BA45/BA46), 5 (located in the right Broca’s area/DLPFC, BA45/BA46), 6 (BA10/BA46), 7 (BA10/BA46), 15 (BA10/BA11), 17 (BA45/BA46), 18 (BA10/BA46/BA47), and 19 (BA45/BA46) in the evaluation period in comparison of after and before negative feedback (Figure 5J). No significant channels were found in other tasks in the DS and PS conditions. *T*-test results are shown in Supplementary Table 6.

### Oxygenated hemoglobin changes in each channel under conditions

**FIGURE 6:**
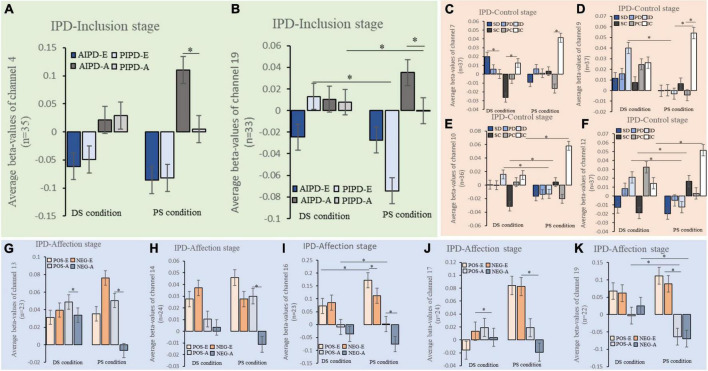
Beta value difference of Oxy-Hb changes in conditions and tasks of each IPD stage. **(A,B)** Differences of the Oxy-Hb changes in conditions and tasks of channels 4 and 19 in the IPD-inclusion stage. **(C–F)** Simple effects analysis results in channels 7, 9, 10, and 12 in the IPD-control stage. **(G–K)** Δbeta-values of Oxy-Hb differences in conditions and tasks of channels 13, 14, 16, 17, and 19 in the IPD-affection stage. DS, differing social status; PS, peer social status; AIPD-E, active interpersonal distance in evaluation periods; PIPD-E, passive interpersonal distance in evaluation periods; AIPD-A, active interpersonal distance in action periods; PIPD-A, passive interpersonal distance in action periods. SD stands for social IPD (1.2 m) under deprivation processes; PD means personal IPD (0.6 m) under deprivation processes; ID means intimate IPD (0.3 m) under deprivation processes. SC stands for social IPD (1.2 m) under controllable processes; PC stands for personal IPD (0.6 m) under controllable processes; IC stands for intimate IPD (0.3 m) under controllable processes. POS-E means positive IPD feedback in evaluation periods. NEG-E means negative IPD feedback in evaluation periods. POS-A means positive IPD feedback in actions. NEG-A means negative IPD feedback in action periods. The column shows the mean beta-values/Δ beta-values in each channel, and the error bar shows the standard error. *p ≤ 0.05.

Interpersonal distance-inclusion stage: ANOVA results showed a main effect of social status for channel 9 (BA10/BA11); the *post-hoc* test revealed more Oxy-Hb changes in the DC condition than in the PS condition (Supplementary Table 4). Moreover, ANOVAs showed the main effect of IPDs for channels 7 (BA10/BA46), 8 (located in the right DLPFC/FPA, BA9/BA10), 11 (BA9/BA10), 12 (BA10), 15 (BA10/BA11), and 18 (BA10/BA46/BA47); the *post-hoc* test reported more Oxy-Hb changes in the PIPD than in the AIPD in the significant channels. In addition, significant main effects in the periods were shown in channels 4 (BA45/BA46), 6 (BA10/BA46), 7 (BA10/BA46), and 11 (BA9/BA10); the *post-hoc* test showed greater Oxy-Hb changes in action period than in the evaluation period in the significant channels. Importantly, interaction effects between social status and IPD were observed in channels 4 (BA45/BA46) and 9 (BA10/BA11) (Figures 6A,B); simple effects analysis showed significant Oxy-Hb changes in the AIPD than in the PIPD of the PS condition, both in channels 4 (BA45/BA46) and 19 (BA45/BA46); simple effects analysis also revealed greater Oxy-Hb changes of PIPD in the DS condition than in the PS condition in channel 19 (BA45/BA46). No other interaction effects were found. Repeated measure ANOVA results of the IPD-Inclusion stage are shown in Supplementary Table 7.

Interpersonal distance-control stage: the main effect of social status was shown in channel 13 (BA10); the *post-hoc* test showed more Oxy-Hb changes in the PS condition than in the DS condition. The main effects of processes were found in channels 4 (BA45/BA46), 6 (BA10/BA46), 17 (BA45/BA46), and 19 (BA45/BA46); the *post-hoc* test reported greater Oxy-Hb changes in the deprivation process than in the controllable process. Moreover, main effects of IPDs showed in channels 5 (BA45/BA46), 7 (BA10/BA46), 8 (BA9/BA10), 9 (BA10/BA11), 10 (located in the FPA, BA10), 11 (BA9/BA10), 12 (BA10), 13 (BA10), 14 (BA9/BA10), and 15 (BA10/BA11); the *post-hoc* test showed greater Oxy-Hb changes in intimate IPD (0.3 m) than in the social IPD (1.2 m) or personal IPD (0.6 m) in the significant channels.

Additionally, interaction effects of social status and processes were shown in channels 10 (BA10), 12 (BA10), 13 (BA10), 14 (BA9/BA10/BA46), 15 (BA10/BA11), 16 (BA10/BA46), 17 (BA45/BA46), and 18 (BA10/BA46/BA47); simple effects analysis showed more Oxy-Hb changes of deprivation process in the DS condition than in the PS condition in channels 10 (BA10), 12 (BA10); simple effects analysis also showed greater Oxy-Hb changes of controllable process in the PS condition than in the DS condition in channels 13 (BA10), 14 (BA9/BA10/BA46), 16 (BA10/BA46), 17 (BA45/BA46), and 18 (BA10/BA46/BA47); simple effects analysis further revealed significant Oxy-Hb changes in the deprivation process than the controllable process, under the DS condition. Furthermore, significant interaction effects of processes and IPDs were observed in channels 4 (BA45/BA46), 5 (BA45/BA46), 7 (BA10/BA46), and 10 (located in the FPA, BA10); simple effects analysis showed more Oxy-Hb changes in the deprivation process than in the controllable process, in the social (1.2 m), personal (0.6 m), and intimate (0.3 m) IPDs of channel 4 (BA45/BA46); simple effects analysis also showed greater Oxy-Hb changes in the intimate IPD (0.3 m) than in the social (1.2 m) or personal (0.6 m) IPDs during the controllable process in channels 5 (BA45/BA46), 7 (BA10/BA46), and 10 (BA10). No interaction effects of social status and IPDs were found.

In addition, significant three-factor interaction effects among social status, processes, and IPDs were shown in channels 7 (lBA10/BA46), 9 (BA10/BA11), 10 (FPA, BA10), and 12 (BA10) (Figures 6C–F). The main results of simple effects analysis showed greater Oxy-Hb changes in the DS condition than in the PS condition during the intimate IPD (0.3 m) deprivation process in channels 9 (BA10/BA11), 10 (BA10), and 12 (BA10). However, simple effects analysis observed more Oxy-Hb changes in the PS condition than in the DS condition in the social (1.2 m) and intimate (0.3 m) IPDs during the controllable process in channels 10 (BA10) and 12 (BA10). Repeated measure ANOVA results of the IPD-Control stage are shown in Supplementary Table 8.

Interpersonal distance-affection stage: the significant main effect of social status showed in the [Oxy-Hb] for channel 17 (BA45/BA46); the *post-hoc* test reported greater [Oxy-Hb] in the PS condition than in the DS condition. Moreover, ANOVA results revealed a significant main effect of IPD feedback for channels 8 (BA9/BA10/BA46), 9 (BA10/BA11), 10 (BA10), 11 (BA9/BA10), 12 (BA10), and 16 (BA10/BA46); the *post-hoc* test showed more [Oxy-Hb] in the positive feedback than negative feedback in all significant channels. Results also found main effects in periods for channels 4 (BA45/BA46), 5 (BA45/BA46), 6 (BA10/BA46), 7 (BA10/BA46), 16 (BA10/BA46), 18 (BA10/BA46/BA47), and 19 (BA45/BA46); the *post-hoc* test showed more [Oxy-Hb] in the evaluation period than in the action period for significant channels.

Besides, ANOVA results showed interaction effects between social status and IPD feedback for channels 14 (BA9/BA10/BA46) and 16 (BA10/BA46) (Figures 6H,I); simple effects analysis showed a significant larger [Oxy-Hb] in the positive feedback than in the negative feedback of the PS condition in channels 14 (BA9/BA10/BA46) and 16 (BA10/BA46); simple effect also showed more [Oxy-Hb] in the PS condition than in the DS condition in the positive feedback for channel 16 (BA10/BA46). Moreover, results showed significant interaction effects in social status and periods for channel 19 (BA45/BA46) (Figure 6K); simple effects analysis showed [Oxy-Hb] in the DS condition were more than in the PS condition during the action period. Results also showed interaction effects of IPD feedback and period for channels 13 (BA10) and 17 (BA45/BA46) (Figures 6G,J); simple effects analysis showed more [Oxy-Hb] in the positive feedback than in the negative feedback during the action period; simple effects analysis also showed greater [Oxy-Hb] of negative feedback in the evaluation period than in the action period. No interaction effects of social status, IPD feedback, and periods were found. Repeated measure ANOVA results of the IPD-Affection stage are shown in Supplementary Table 9.

### Correlation between oxygenated hemoglobin changes and interpersonal distance performance

**FIGURE 7:**
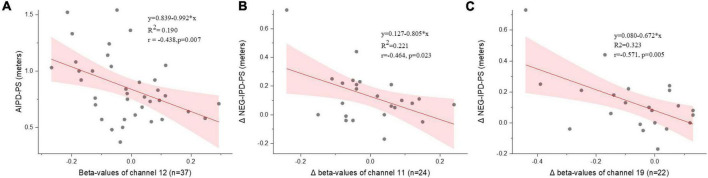
Correlation between IPD performance and beta values of Oxy-Hb changes in the IPD-inclusion stage and IPD-Affection stage. **(A)** A significant correlation between changes of Oxy-Hb in channel 12 and the AIPD during the evaluation period in the PS condition. **(B,C)** A significant correlation between Δbeta-values of Oxy-Hb changes in channels 11 and 19 and the ΔNEG-IPD during the action period in the PS condition. PS, peer social status; AIPD, active interpersonal distance; ΔNEG-IPD, IPD gaps in IPD after negative feedback minus the baseline; Each scatter plot reports the equation formula (y = b + a*x), regression square of fitted linear regression (*R*^2^), Pearson correlation coefficients (r), *p*-values, 95% confidence interval (pink shadow) and one piece of dyad data (gray dot).

Significant negative correlations were observed in the IPD-Inclusion and the IPD-Affection stages in the PS condition only. In the IPD-Inclusion stage, a negative correlation was found between AIPD performance and Oxy-Hb changes in channel 12 (BA10) during the evaluation period (*r* = −0.438, *p* = 0.007) (Figure 7A). In the IPD-Affection stage, negative correlation between ΔNEG-IPD (after negative feedback and baseline) and [Oxy-Hb] in channels 11 (BA9/BA10) (*r* = −0.464, *p* = 0.023) and 19 (BA45/BA46) (*r* = −0.571, *p* = 0.005) during the action period (Figures 7B,C). No other significant correlations were found.

## Discussion

The present research investigated IPD performance and PFC alterations in the development process of real-time differing social status IPD interactions for the first time, combining objectives from social-behavioral psychology and neuroscience. We explored PFC alterations based on modified stop-distance paradigms using fNIRS in healthy participants. We observed a significantly larger IPD in the DS condition than in the PS condition in the IPD-Inclusion stage. The participants did not tend to get close to the teachers but were closer to the students with IPD development (IPD-Control and IPD-Affection stages). Importantly, FPA (BA10), DLPFC (BA9/BA46), and Broca’s area (BA45) activations in real-time differing social status IPD interactions were consistently found along with IPD development for the first time. We found brain activations both in the evaluation and action period in the IPD-Inclusion stage; enhanced FPA (BA10) in the PIPD-E both in the DS and PS conditions; and FPA (BA10), DLPFC (BA9/BA45), and Broca’s area (BA45) responded to the IPD actions. In addition, activated FPA (BA10) and DLPFC (BA9/BA46) in the intimated IPD (0.3 m) were shown in the IPD-Control stage. In the IPD-Affection stage, significant enhancement of the FPA (BA10), DLPFC (BA9/BA46), and Broca’s area (BA45) was observed during the evaluation period after positive and negative feedback both in the DS and PS conditions. Notably, significant differences Oxy-Hb changes in channels located in the Broca’s area (BA45), FPA (BA10), and DLPFC (BA9/BA46) were observed between the DS condition and PS condition in the three IPD stages. Accordingly, the results suggest that the differing social status affects IPD performance and that FPA (BA10), DLPFC (BA9/BA46), and Broca’s area (BA45) play critical roles during the real-time differing social status IPD interactions in different stages.

### Interpersonal distance gaps in real-time differing social status interpersonal distance interactions

We found that IPD gaps exist between differing social status. Participants maintained a larger IPD with teachers than with peer students, which is supported by the IPD-Inclusion stage. Previous studies have shown that social status strongly affects IPD (Gifford, 1982); our study supports this view. In addition, the participants remained at a distance from the teachers in the IPD-Control stage but were willing to approach peer students. In the subjective IPD questionnaire, the DS condition did not change, whereas the PS condition showed that 25 participants selected personal IPD (0.6 m) as the most comfortable distance. Furthermore, IPD action (after feedback) showed consistent results with prior social feedback studies, with a closer IPD after positive feedback and a larger IPD following negative feedback (Knowles et al., 2014; Johnson, 2016). However, the IPD changes after IPD feedback in the DS condition were not as pronounced as those in the PS condition, implying that participants preferred to maintain a relatively stable and safe IPD from the teacher. Accordingly, the results indicate that there are IPD gaps between differing social status, and student participants tended to maintain a spatial IPD from the teacher while developing an intimate IPD toward other students.

### Brain activations in real-time differing social status interpersonal distance interactions

The brain activations in the differing social status IPD interaction showed stage features. In the IPD-Inclusion stage, the present study revealed brain activations in IPD evaluation and action periods, indicating that participants focused on both IPD cognitions at the beginning stage. Moreover, results in the IPD-Control stage indicated that FPA (BA10) and DLPFC (BA9/BA46) changes were sensitive to intimate IPD (0.3 m) in the deprivation (DS condition) and controllable (PS condition) process. It indicated the function of PFC in IPD motion sensitivity and control; a narrow IPD evokes PFC changes. Additionally, the activated FPA (BA10), DLPFC (BA9/BA46), and Broca’s area (BA45) was shown both in the DS and PS conditions during the evaluation periods after receiving the positive and negative IPD feedback (compared to baseline) in the IPD-Affection stage. fMRI studies showed that social evaluation feedback aroused DLPFC (BA9/BA46) and MPFC (Achterberg et al., 2020) and demonstrated the role of DLPFC (BA9/BA46) in processing social hierarchy (Zink et al., 2008). Although Broca’s area was not the ROI initially, it was consistently active in IPD interactions. The Broca’s area was proved as a critical region for social communication, including the involvement of non-verbal behaviors (Hupfeld et al., 2017) and sign language (Trettenbrein et al., 2021). Our results highlighted the function of FPA (BA10), DLPFC (BA9/BA46), and Broca’s area (BA45) in real-time differing social status IPD interactions after social feedback. Therefore, FPA (BA10), DLPFC (BA9/BA46), and Broca’s area (BA45) cognitive resources focus on different IPD cognitive tasks at different stages and are influenced by IPD stages. In short, brain activities shifted along with IPD development in the differing social status interactions.

Furthermore, greater Oxy-Hb changes in the FPA (BA10) and DLPFC (BA9/BA46) showed in PIPD relative to AIPD both in the evaluation and action period of the IPD-Inclusion stage. Neural studies showed a significant alteration in the DLPFC (BA9/BA46) and MPFC in approaching social tasks (Schienle et al., 2017; Cohen and Shamay-Tsoory, 2018), which is similar to the PIPD task in the present study. However, those studies did not discuss the brain activities in the AIPD and the neural differences between the AIPD and PIPD. PIPD is a social distance passively approached by others, which may violate interpersonal spatial boundaries. Participants’ PFC activations concentrated on processing PIPD may be because PIPD is associated with self-space protection and prevention of aggression by others, which elicits self-boundary vigilance. Previous studies showed the significant role of the amygdala in the PIPD cognitions (Wabnegger et al., 2016). The amygdala functions in dealing with social threats and fear (Twining et al., 2017), and evidence indicated DLPFC (BA9/BA46) connectivity with the amygdala during threat processing (Gold et al., 2015). Our results supported the amygdala-prefrontal neural circuit. Besides, the IPD interaction in the first stage may cause social anxiety. An fNIRS study showed that the FPA (BA10) reflects social anxiety in a free speaking task (Tomita and Kumano, 2021). Accordingly, the present study proposed that FPA (BA10) and DLPFC (BA9/BA46) play a critical role in PIPD interactions at the beginning of the IPD interaction for the self-protective.

Additionally, action and non-action period comparisons showed brain activations in each differing social status IPD stage. Results in comparison of evaluation and action periods indicated the distinguished brain function in processing IPD tasks. Compared to the evaluation, the action period showed significant activities in Broca’s area (BA45) and DLPFC (BA9/BA46). But enhanced Oxy-Hb changes in FPA (BA10) were observed in the evaluation period relative to the action period. DLPFC has been regarded as an essential brain area in motor behavior (Cieslik et al., 2013), and FPA (BA10) affects the decision-making efficacy (Laureiro-Martínez et al., 2014). What we found in the present study highlighted Broca’s area (BA45) and DLPFC (BA9/BA46) functions in social actions and FPA (BA10) in social evaluations. In addition, we confirmed the significant Oxy-Hb changes in the deprivation (non-action) relative to controllable (action) processes in the Broca’s area (BA45), DLPFC (BA9/BA46), and FPA (BA10). It indicated that being approached by others in different IPDs consumed more resources than taking IPD actions. However, things are complicated if we consider social status; we discussed this point in 4.3.

Differences between non-action relative to action IPD process were also found in the IPD-Affection stage; DLPFC (BA9/BA46), FPA (BA10), and Broca’s area (BA45) only showed in the evaluation period (after feedback) compared to baseline (before feedback), implying a complex social interaction context aroused significant brain activations. However, the IPD feedback from teacher/peer students significantly impacted the IPD evaluation even though participants were aware of their most comfortable IPD. It implies the feedback effects in interpersonal interactions. From one aspect, deciding the most comfortable IPD became a complex process after IPD feedback. Neuroimage studies propose the vital role of right-DLPFC (BA9/BA46) in social interactions, such as in the decision-making of ultimatum game (Speitel et al., 2019), and significant IBS shown in the right-DLPFC (BA9/BA46) in the group decision-making behavior (Zhang et al., 2021). From another aspect, the IPD action after feedback is the outcome of the self-IPD and the IPD of partners (teacher/peer student). Lesions in FPA (BA10) were shown to be dysfunctional in prosocial behavior (Moll et al., 2011). Evidence indicated that FPA (BA10) is significant in self-other social interactions (Ruby and Decety, 2004). Besides, functions of Broca’s area (BA45) have been expanded beyond verbal cognition, such as motion observation (Caspers et al., 2010) and involvement in cooperation (Zhou et al., 2022). Our results further enrich the social functions of the Broca’s area (BA45). Results in the third IPD stage demonstrated the role of brain alterations in IPD evaluation following social feedback. Therefore, the current study indicated the significance and flexibility of the FPA (BA10), DLPFC (BA9/BA46), and Broca’s area (BA10) in dealing with different IPD tasks in different IPD stages.

The present research represents the first step in proving that the DLPFC (BA9/BA46), FPA (BA10), and Broca’s area (BA45) functions under rich real-time IPD interactions in differing social status in each IPD stage. The DLPFC (BA9/BA46) is considered to be a social brain specific to social cognition and control (Schmidt et al., 2018) and is related to social affective and sensory processing (Burns, 2006). We observed the DLPFC (BA9/BA46) activation in IPD cognitions across three IPD stages. Moreover, the FPA (BA10) has been implicated in the representation of realistic social interactions, like face-to-face conversations (Suda et al., 2010). Here, the role of the FPA (BA10) was highlighted in the IPD evaluation and action task periods. Besides, the present study uncovered the participation of Broca’s area in non-verbal IPD interactions and added new insight into the role of Broca’s area as a sophisticated brain region. Therefore, our findings support and highlight the role of the DLPFC (BA9/BA46), FPA (BA10), and Broca’s area (BA45) during real-time IPD interactions between differing social status.

### Differences in neural changes between the differing social status condition and peer social status condition

The present study identified significant differences in neural changes between the DS condition and the PS condition. In the IPD-Inclusion stage, greater neural changes in the DS condition than in the PS condition in PIPDs. It indicated that IPD interactions with the teacher are an energy-consuming and stressful social situation for the student participants, especially in a passive status. However, we only found neural activations during IPD action periods in the PS conditions. It is suggested that interacting with a peer student is more intreating for student participants compared to teachers.

In the IPD-Control stage, simple effects analysis results indicated a neural resource-consuming under deprivation process, especially when approached by teachers arrived at an intimate IPD (0.3 m). This finding suggested the social avoidance of participants from teachers, which is also supported by the evidence from the controllable process. We found the neural changes actively in the controllable process of the peers relative to teachers, indicating the willingness, interest, and tendency to establish a relationship with peers. Claims based on the neuro changes are consistent with the behavior and questionnaire results. We proposed that social status affects the Oxy-Hb changes to different IPD processes in the differing social status IPD interactions.

In the IPD-Affection stage, we observed more Oxy-Hb differences before and after positive feedback in the PS condition than in the DS condition. After participants received IPD feedback, they actively considered and acted to the IPD between themselves and peers, reflected in a significant increase in the PS condition relative to the DS condition during the IPD interactions after positive feedback. It implies that the feedback effects are more meaningful in the PS condition relative to the DS condition. However, greater neural changes in the DS condition than in the PS condition were observed after feedback during the action periods. It may activate plenty of cognition resources to take action after feedback from teachers, as they are of high status and always an authority to student participants.

Our results are partly consistent with neural findings in other status-gap social interactions. One study showed that cooperating with a teacher consumes more neural changes than cooperating with a student (Sun et al., 2021). Communications in leader-follower pairs revealed more neural activity than in follower–follower pairs (Jiang et al., 2015). However, the present study found more neural changes in the PS condition relative to the DS condition in some IPD interaction contexts. It means that IPD interactions between differing social status are incredibly complex, and the cognitive processing of IPD interactions changes depending on the interaction situation between differing social status. Furthermore, significant differences in neural changes in social status were observed in the Broca’s area (BA45), FPA (BA10), and DLPFC (BA9/BA46). Neuroimage evidence showed significant differences in the Broca’s area (BA45) and DLPFC (BA9/BA46) between low- and high-socioeconomic disparity groups in a live pro-social dialog (Descorbeth et al., 2020). Hence, the present study proposed the critical role of Broca’s area (BA45), FPA (BA10), and DLPFC (BA9/BA46) in processing differing social status under real-time IPD social interaction, and further research is needed.

### Correlations between interpersonal distance performance and neural changes

We found negative correlations between IPD performance and neural changes only during evaluation and action periods in the PS condition. The results are partly consistent with previous studies. Connectivity strength between the midbrain and the left premotor cortex negatively correlated with the IPD performance (Vieira et al., 2020). Meanwhile, other fMRI studies showed positive correlations between the dorsal intraparietal sulcus and IPD performance (Holt et al., 2015). The significant negative correlations between neural changes and IPD performance in the present study could be a reference for real-time IPD studies. Furthermore, correlations showed both in the IPD-Inclusion and IPD-Affection stage. In the IPD-Inclusion stage, the negative correlations indicated that the more neural changes, the smaller the AIPD performance is. It means achieving a close IPD accompanied by numerous PFC changes. In the IPD-Affection stage, the more neural changes between after and before negative feedback, the less alter in IPD after feedback. It implied that the less the IPD changes, the more the differences in Oxy-Hb change before and after negative feedback, which means keeping the initial IPD for oneself arouses the neural changes. Moreover, the negative correlations were located in the FPA (BA10) during the evaluation period, but Broca’s area (BA45) and DLPFC (BA9/BA46) in the action periods. It may imply FPA (BA10) sensitivities to consideration cognition process and the Broca’s area (BA45) and DLPFC (BA9/BA46) response to the IPD actions. Thus, we proposed that Oxy-Hb changes in the FPA (BA10), Broca’s area (BA45), and DLPFC (BA9/BA46) reflect IPD performance in real-time differing social status IPD interactions, and additional research is needed.

### Limitations and future directions

The present study has some limitations that we must consider. First, there are limitations to the three IPD stages. The reaction of the action period at the stop time points of participants varied as the step speed of each participant was different. Although this did not seem to affect the final results, a more consistent experimental design is needed to improve the precision of future research. In addition, the present study has limitations with respect to the specific IPD stage. During the IPD-Control stage, the order of deprivation and control conditions was not balanced. This may have resulted in a sequential effect. This problem should be addressed in future studies. In the IPD-Affection stage, we performed an ecological and natural IPD interaction using the dynamic IPD paradigm. However, some participants were excluded because of failure in the experiment performance. One reason is the strict inclusion criteria; once the paradigm in one condition failed, the data were rejected from the activation analysis. Another reason is that the experiment was conducted during the COVID-19 pandemic and was stopped several times to prevent the spread of the virus and then restarted. This affected the continuity of the distance experience of the experimental supporters (teachers/peer students) and the correctness of the IPD feedback. Although the experiment faced many obstacles, it recruited sufficient sample data to report brain activation during IPD interaction. Lastly, the present study focused on the PFC area only, which limited the opportunity to enrich the brain activity evidence, like the parietal and temporal cortex, in the real-time IPD interactions. Future studies should expand the ROIs in the real-time IPD interactions and include multiple brain regions with advanced fNIRS devices.

In future research, we would like to offer some proposals. First, the ecological IPD paradigms in the present study could be applied to other social contexts. The present study did not set a specific social situation, while IPD interactions occur across various daily backgrounds, for example, the IPD may vary in a conversation or a meeting room. Second, more efforts are needed to understand IPD performance and neural alterations. Although the present study found a significant relationship between these variables, it showed a relatively weak correlation. The cognitive processing of IPD in differing social status is complex, and future research could diversify the assessment of IPD behavioral indicators by, for example, combining the assessment of IPD reaction time. Alternatively, future studies could also extend the brain regions of interest to further verify the relationship between variations in brain activity and IPD performance in differing social status IPD interactions. Lastly, while differing social status interaction is a two-person issue, the current study was conducted from one person’s perspective. A neural study of both people should be undertaken and the most comfortable IPD that is accepted by each other should be measured in future studies based on new IPD interaction paradigms.

## Conclusion

The present fNIRS study provides the first examination of differing social status IPD interactions in the development process. Based on FIRO theory, we conducted three IPD experiments corresponding to the inclusion, control, and affective stages of IPD interactions. We found that the FPA (BA10), DLPFC (BA9/BA46), and Broca’s area (BA45) were recruited to support the IPD interaction. The present study demonstrated for the first time the changes in PFC and Broca’s area that accompany the development process of differing social status IPD interactions. We uncovered the functional activation of the FPA (BA10), DLPFC (BA9/BA46), and Broca’s area (BA45) for IPD evaluation and action. We identified the critical role of the FPA (BA10), DLPFC (BA9/BA46), and Broca’s area (BA45) in the IPD evaluation period after feedback. In addition, FPA (BA10) and DLPFC (BA9/BA46) were sensitive to intimate IPD (0.3 m) in both conditions, significant during the deprivation process in the DS condition but noticeable in the controllable process in the PS condition. Furthermore, we demonstrated that different social effects in the Broca’s area (BA45), FPA (BA10), and DLPFC (BA9/BA46) varied according to the IPD interaction situations between the DS and PS conditions, which supports the function of those brain areas in social status processing under non-verbal interactions. The current study provides insight into the negative correlation between Oxy-Hb changes of the FPA (BA10), DLPFC (BA9/BA46), and Broca’s area (BA45) and IPD performance, indicating the PFC and Broca’s area (BA45) sensitivity for IPD cognition. Additionally, this study offers experimental paradigms for future real-time human IPD interaction research as a reference. Notably, the present study proves that neural alterations in differing social status IPD interactions from a systematic perspective deepen the comprehension of differing social status non-verbal behavior interactions.

## Data availability statement

The raw data supporting the conclusions of this article will be made available by the authors, without undue reservation.

## Ethics statement

The studies involving human participants were reviewed and approved by the Ethical Committee of Tohoku University (Sendai, Japan). The patients/participants provided their written informed consent to participate in this study.

## Author contributions

XH and S-II contributed to conceptualization and writing—review and editing. XH contributed to experimental execution and writing—original draft preparation. XH and YS contributed to data analysis. S-II contributed to supervision. All authors contributed to the article and approved the submitted version.

## Footnotes

1
https://www.alivelearn.net/xjview

## Abbreviations

IPD: interpersonal distance
DS condition: differing social status condition
PS condition: peer social status condition
fNIRS: functional near-infrared spectroscopy
Oxy-Hb: oxyhemoglobin
FPA: frontopolar area
DLPFC: dorsolateral prefrontal cortex
PFC: prefrontal cortex.
