# Supplementary material for: Neural and behavioral alterations of a real-time interpersonal distance (IPD) development process in differing social status interactions
Source: Front Behav Neurosci. 2022 Oct 13;16:969440. doi: 10.3389/fnbeh.2022.969440 (PMC9616044; doi:10.3389/fnbeh.2022.969440)
Supplement: Supplementary file 1 [file Data_Sheet_1.docx]

Supplementary Material

# Supplementary Tables

**Table 1: The registration locations on the MNI of each channel**

| **Channels** | **MNI (x y z)** | **Activation Brain regions** | **BA** | **Overlap (%)** |
| --- | --- | --- | --- | --- |
| Channel 4 | 55.4 38.4 11.8 | pars triangularis Broca's area  Dorsolateral prefrontal cortex | 45  46 | 97  3 |
| Channel 5 | 47.4 44.6 26.4 | pars triangularis Broca's area  Dorsolateral prefrontal cortex | 45  46 | 72  28 |
| Channel 6 | 47.3 54.4 1.6 | Frontopolar area  Dorsolateral prefrontal cortex | 10  46 | 15  85 |
| Channel 7 | 38.3 59.5 16.5 | Frontopolar area  Dorsolateral prefrontal cortex | 10  46 | 46  54 |
| Channel 8 | 26.9 59.7 29.9 | Dorsolateral prefrontal cortex  Frontopolar area  Dorsolateral prefrontal cortex | 9  10  46 | 20  30  50 |
| Channel 9 | 27.4 69.1 5.6 | Frontopolar area  Orbitofrontal area | 10  11 | 72  28 |
| Channel 10 | 16.9 69.1 19 | Frontopolar area | 10 | 100 |
| Channel 11 | 4.3 63.6 30.4 | Dorsolateral prefrontal cortex  Frontopolar area | 9  10 | 18  82 |
| Channel 12 | 3.8 71.6 5.9 | Frontopolar area | 10 | 100 |
| Channel 13 | -11 69.4 18.1 | Frontopolar area | 10 | 100 |
| Channel 14 | -21.5 61.6 29 | Dorsolateral prefrontal cortex  Frontopolar area  Dorsolateral prefrontal cortex | 9  10  46 | 18  48  34 |
| Channel 15 | -22.1 69.9 4.1 | Frontopolar area  Orbitofrontal area | 10  11 | 68  32 |
| Channel 16 | -34.6 61.4 14.4 | Frontopolar area  Dorsolateral prefrontal cortex | 10  46 | 59  41 |
| Channel 17 | -44.1 47.2 24.4 | pars triangularis Broca's area  Dorsolateral prefrontal cortex | 45  46 | 55  45 |
|  |  |  |  |  |
| Channel 18 | -43.8 55.7 -1 | Frontopolar area  Dorsolateral prefrontal cortex  Inferior prefrontal gyrus | 10  46  47 | 24  75  1 |
| Channel 19 | -52.4 40.6 8.5 | pars triangularis Broca's area  Dorsolateral prefrontal cortex | 45  46 | 85  15 |

MNI, Montreal Neurological Institute; BA, Brodmann Area;

**Table 2: The valid fNIRS data in three experiments in group-analysis of activation and each channel analysis**

| N=38 (Total) | Experiment 1 | |  | Experiment 2 | |  | Experiment 3 | |
| --- | --- | --- | --- | --- | --- | --- | --- | --- |
|  | Excluded | Valid data |  | Excluded | Valid data |  | Excluded | Valid data |
| Group-analysis | 2 | 36 |  | 1 | 37 |  | DS condition: 11  PS condition: 7 | DS condition: 27  PS condition: 31 |
| Channel 4 | 4 | 34 |  | 3 | 35 |  | 9 | 29 |
| Channel 5 | 2 | 36 |  | 1 | 37 |  | 7 | 31 |
| Channel 6 | 2 | 36 |  | 1 | 37 |  | 7 | 31 |
| Channel 7 | 2 | 36 |  | 1 | 37 |  | 7 | 31 |
| Channel 8 | 8 | 30 |  | 7 | 33 |  | 12 | 26 |
| Channel 9 | 3 | 35 |  | 2 | 37 |  | 7 | 31 |
| Channel 10 | 2 | 36 |  |  |  |  | 8 | 30 |
| Channel 11 | 2 | 36 |  | 1 | 37 |  | 7 | 31 |
| Channel 12 | 2 | 36 |  | 1 | 37 |  | 7 | 31 |
| Channel 13 | 2 | 36 |  | 2 | 36 |  | 7 | 31 |
| Channel 14 | 2 | 36 |  | 1 | 35 |  | 7 | 31 |
| Channel 15 | 3 | 35 |  | 2 | 36 |  | 8 | 30 |
| Channel 16 | 2 | 36 |  | 2 | 36 |  | 8 | 30 |
| Channel 17 | 2 | 36 |  | 1 | 37 |  | 7 | 31 |
| Channel 18 | 2 | 36 |  | 1 | 37 |  | 7 | 31 |
| Channel 19 | 6 | 32 |  | 5 | 34 |  | 11 | 27 |

| N = 35 (valid) | Social IPD  (1.2 meters) | Personal IPD  (0.6 meters) | Intimate IPD  (0.3 meters) | Total  (N) | χ2 | p-value |
| --- | --- | --- | --- | --- | --- | --- |
| DS-deprivation | 20 | 15 | 0 | 35 | 0.714 | 0.398 |
| DS-control | 18 | 17 | 0 | 35 | 0.029 | 0.866 |
| PS-deprivation | 15 | 20 | 0 | 35 | 0.714 | 0.398 |
| PS-control | 8 | 25 | 2 | 35 | 24.400 | 0.000 |

**Table 3: The Chi-square results of question choice under conditions in experiment 2**

DS-deprivation, The IPD-deprivation process in the differing social status condition. DS-control, The IPD-control process in the differing social status condition. PS-deprivation, The IPD-deprivation process in the peer social status condition. PS-control, The IPD-control process in the peer social status condition.

**Table 4: Activation channels in the IPD-Inclusion stage**

|  | **DS-AIPD-E** | | **DS-PIPD-E** | | **DS-AIPD-A** | | **DS-PIPD-A** | | **PS-AIPD-E** | | **PS-PIPD-E** | | **PS-AIPD-A** | | **PS-PIPD-A** | |
| --- | --- | --- | --- | --- | --- | --- | --- | --- | --- | --- | --- | --- | --- | --- | --- | --- |
| **Channels** | **t** | **p** | **t** | **p** | **t** | **p** | **t** | **p** | **t** | **p** | **t** | **p** | **t** | **p** | **t** | **p** |
| Channel 4 | -2.123 | 0.134 | -2.068 | 0.128 | 0.906 | 0.279 | 0.999 | 0.266 | -3.556 | 0.014 | -3.053 | 0.032 | 2.857 | 0.043 | 0.192 | 0.417 |
| Channel 5 | 1.850 | 0.129 | 0.993 | 0.263 | -0.395 | 0.379 | 1.232 | 0.233 | -1.322 | 0.212 | 0.218 | 0.419 | 0.87 | 0.285 | 1.52 | 0.195 |
| Channel 6 | -1.216 | 0.234 | -0.624 | 0.339 | 1.431 | 0.199 | 2.053 | 0.124 | -2.655 | 0.061 | -1.088 | 0.250 | 1.612 | 0.184 | 1.498 | 0.185 |
| Channel 7 | 0.124 | 0.421 | -0.582 | 0.344 | 1.421 | 0.198 | 2.459 | 0.088 | -1.974 | 0.130 | -0.159 | 0.423 | 1.015 | 0.263 | 3.235 | 0.028 |
| Channel 8 | -0.665 | 0.338 | 1.439 | 0.202 | -1.139 | 0.240 | -0.238 | 0.418 | -1.353 | 0.211 | 0.689 | 0.332 | -0.232 | 0.417 | 0.576 | 0.344 |
| Channel 9 | -1.501 | 0.192 | 0.873 | 0.288 | 1.168 | 0.239 | 1.953 | 0.131 | -1.308 | 0.213 | -0.124 | 0.414 | 0.153 | 0.421 | 0.937 | 0.270 |
| Channel 10 | 0.827 | 0.295 | 1.58 | 0.190 | -0.203 | 0.417 | 0.141 | 0.422 | -0.39 | 0.377 | 0.742 | 0.322 | 0.513 | 0.354 | 0.621 | 0.336 |
| Channel 11 | 1.984 | 0.133 | 3.456 | 0.028 | -0.986 | 0.263 | -0.972 | 0.264 | -0.125 | 0.418 | 2.899 | 0.042 | -1.033 | 0.259 | -1.071 | 0.249 |
| Channel 12 | -0.088 | 0.417 | 1.126 | 0.240 | 0.422 | 0.372 | 2.244 | 0.108 | -0.742 | 0.326 | 0.179 | 0.419 | 0.658 | 0.337 | 1.743 | 0.152 |
| Channel 13 | 1.615 | 0.188 | 1.88 | 0.130 | -1.508 | 0.196 | -0.429 | 0.372 | -0.518 | 0.356 | 1.261 | 0.226 | -0.638 | 0.340 | -0.004 | 0.440 |
| Channel 14 | 0.474 | 0.358 | 2.664 | 0.067 | -1.082 | 0.249 | -0.561 | 0.342 | -0.002 | 0.433 | 1.503 | 0.188 | -0.139 | 0.419 | -2.089 | 0.136 |
| Channel 15 | -1.331 | 0.213 | 0.636 | 0.338 | 0.948 | 0.269 | 2.299 | 0.104 | -1.55 | 0.195 | 0.066 | 0.421 | 0.218 | 0.415 | 2.419 | 0.090 |
| Channel 16 | 0.727 | 0.324 | 0.368 | 0.382 | 1.537 | 0.194 | 1.799 | 0.141 | -0.486 | 0.357 | -0.114 | 0.411 | 1.888 | 0.133 | 2.093 | 0.129 |
| Channel 17 | 1.99 | 0.136 | 3.549 | 0.019 | -1.156 | 0.236 | -0.844 | 0.291 | 0.359 | 0.381 | 1.191 | 0.239 | -0.715 | 0.325 | -0.565 | 0.344 |
| Channel 18 | -1.864 | 0.130 | 0.123 | 0.411 | 0.597 | 0.342 | 1.404 | 0.196 | -1.421 | 0.194 | -2.372 | 0.091 | 1.943 | 0.128 | 3.706 | 0.011 |
| Channel 19 | -1.179 | 0.241 | 0.498 | 0.356 | 0.331 | 0.389 | 0.288 | 0.402 | -1.165 | 0.238 | -3.931 | 0.000 | 1.95 | 0.123 | -0.004 | 0.436 |

DS-AIPD-E, active interpersonal distance during evaluation periods in the differing social status condition; DS-PIPD-E, passive interpersonal distance during evaluation periods in the differing social status condition; DS-AIPD-A, active interpersonal distance during action periods in the differing social status condition; DS-PIPD-A, active interpersonal distance during action periods in the differing social status condition; PS-AIPD-E, active interpersonal distance during evaluation periods in the peer social status condition; PS-PIPD-E, passive interpersonal distance during evaluation periods in the peer social status condition; PS-AIPD-A, active interpersonal distance during action periods in the peer social status condition; PS-PIPD-A, active interpersonal distance during action periods in the peer social status condition; t, t values; p, adjusted p values by the False Discovery Rate;

**Table 5: Activation channels in the IPD-Control stage**

|  | **DS-SD1** | | **DS-PD1** | | **DS-ID1** | | **PS-SD1** | | **PS-PD1** | | **PS-ID1** | | **DS-SD2** | | **DS-PD2** | | **DS-ID2** | | **PS-SD2** | | **PS-PD2** | | **PS-ID2** | | |
| --- | --- | --- | --- | --- | --- | --- | --- | --- | --- | --- | --- | --- | --- | --- | --- | --- | --- | --- | --- | --- | --- | --- | --- | --- | --- |
| **Channels** | **t** | **p** | **t** | **p** | **t** | **p** | **t** | **p** | **t** | **p** | **t** | **p** | **t** | **p** | **t** | **p** | **t** | **p** | **t** | **p** | **t** | **p** | **t** | **p** |  |
| Channel 4 | -0.768 | 0.339 | 1.891 | 0.122 | 1.332 | 0.213 | -1.000 | 0.268 | 0.695 | 0.347 | 1.152 | 0.243 | -3.720 | 0.023 | -3.936 | 0.000 | -3.376 | 0.017 | -3.164 | 0.021 | -4.447 | 0.000 | -2.332 | 0.061 |  |
| Channel 5 | -0.239 | 0.434 | 0.329 | 0.423 | 0.499 | 0.381 | -0.449 | 0.392 | 0.201 | 0.440 | 0.661 | 0.356 | -3.119 | 0.024 | -0.005 | 0.474 | 1.675 | 0.151 | -0.365 | 0.413 | -1.630 | 0.159 | 2.406 | 0.056 |  |
| Channel 6 | 0.837 | 0.314 | 1.616 | 0.161 | 2.541 | 0.056 | -0.743 | 0.341 | 0.938 | 0.281 | 1.268 | 0.218 | -1.647 | 0.156 | -1.375 | 0.205 | -2.833 | 0.037 | -2.456 | 0.054 | -3.493 | 0.015 | 0.604 | 0.368 |  |
| Channel 7 | 1.862 | 0.127 | 0.554 | 0.379 | -0.001 | 0.473 | -0.751 | 0.343 | 0.638 | 0.363 | 0.167 | 0.441 | -2.392 | 0.057 | -0.311 | 0.428 | 1.081 | 0.253 | 0.251 | 0.437 | -1.241 | 0.225 | 2.219 | 0.071 |  |
| Channel 8 | -0.117 | 0.449 | 0.559 | 0.379 | 1.006 | 0.269 | -0.490 | 0.381 | -1.812 | 0.136 | 0.238 | 0.432 | -1.463 | 0.183 | -0.954 | 0.277 | -0.139 | 0.445 | -0.614 | 0.366 | -1.729 | 0.146 | 1.723 | 0.144 |  |
| Channel 9 | 1.320 | 0.214 | 2.150 | 0.080 | 3.869 | 0.000 | -0.014 | 0.473 | -0.030 | 0.469 | -0.280 | 0.436 | 0.524 | 0.386 | 1.146 | 0.240 | 1.603 | 0.160 | 0.399 | 0.406 | -0.260 | 0.439 | 2.770 | 0.043 |  |
| Channel 10 | 0.085 | 0.458 | -0.067 | 0.463 | 1.493 | 0.179 | -1.615 | 0.158 | -1.484 | 0.178 | -1.485 | 0.179 | -2.552 | 0.055 | 0.248 | 0.434 | 0.625 | 0.364 | 0.280 | 0.433 | -0.933 | 0.277 | 2.455 | 0.052 |  |
| Channel 11 | -1.290 | 0.217 | 1.012 | 0.273 | 2.525 | 0.054 | -2.215 | 0.075 | -0.578 | 0.376 | -1.210 | 0.229 | -2.328 | 0.064 | 1.570 | 0.165 | 0.732 | 0.341 | 0.997 | 0.267 | 0.184 | 0.442 | 2.678 | 0.045 |  |
| Channel 12 | -1.290 | 0.214 | 1.012 | 0.270 | 2.525 | 0.052 | -2.215 | 0.073 | -0.578 | 0.374 | -1.210 | 0.226 | -2.328 | 0.062 | 1.570 | 0.162 | 0.732 | 0.338 | 0.997 | 0.265 | 0.184 | 0.439 | 2.678 | 0.043 |  |
| Channel 13 | -3.422 | 0.023 | -1.303 | 0.218 | 0.705 | 0.344 | -1.171 | 0.238 | -1.280 | 0.216 | -1.009 | 0.269 | -1.958 | 0.110 | -0.503 | 0.382 | -0.142 | 0.446 | 1.140 | 0.238 | 0.459 | 0.391 | 3.135 | 0.019 |  |
| Channel 14 | -2.022 | 0.101 | -1.129 | 0.240 | 1.377 | 0.207 | -0.631 | 0.364 | -0.678 | 0.351 | -0.730 | 0.336 | -3.630 | 0.018 | -1.721 | 0.145 | -0.936 | 0.279 | -0.737 | 0.342 | -0.416 | 0.401 | 2.685 | 0.042 |  |
| Channel 15 | -0.386 | 0.406 | 1.998 | 0.103 | 1.816 | 0.136 | -1.295 | 0.218 | -0.529 | 0.387 | -0.053 | 0.466 | -1.506 | 0.178 | 0.747 | 0.343 | 0.427 | 0.399 | 0.505 | 0.384 | 0.259 | 0.436 | 2.395 | 0.056 |  |
| Channel 16 | 1.533 | 0.172 | 0.292 | 0.433 | 1.683 | 0.151 | -0.521 | 0.385 | 0.248 | 0.436 | 0.515 | 0.382 | -2.732 | 0.045 | -1.229 | 0.227 | -1.220 | 0.228 | -1.106 | 0.246 | 0.187 | 0.443 | 2.149 | 0.081 |  |
| Channel 17 | 0.498 | 0.379 | 0.172 | 0.442 | 1.148 | 0.242 | -1.723 | 0.148 | 1.141 | 0.240 | -0.119 | 0.450 | -3.290 | 0.020 | -1.733 | 0.149 | -2.902 | 0.034 | -0.816 | 0.321 | -2.748 | 0.045 | 1.031 | 0.268 |  |
| Channel 18 | -0.146 | 0.447 | 1.765 | 0.142 | 2.453 | 0.056 | 0.223 | 0.436 | 0.977 | 0.270 | 1.599 | 0.159 | -3.282 | 0.018 | -1.350 | 0.211 | -1.777 | 0.141 | -0.520 | 0.383 | -0.219 | 0.435 | 1.037 | 0.268 |  |
| Channel 19 | 0.356 | 0.414 | -0.034 | 0.471 | 2.526 | 0.053 | -1.402 | 0.201 | -0.396 | 0.405 | 1.340 | 0.213 | -2.494 | 0.055 | -2.722 | 0.043 | -1.929 | 0.115 | -3.292 | 0.015 | -3.664 | 0.013 | -2.154 | 0.079 |  |

DS-SD1 , social interpersonal distance (1.2m) of differing social status during deprivation process; DS-PD1, personal interpersonal distance (0.6m) of differing social status during deprivation process; DS-ID1, intimate interpersonal distance (1.2m) of differing social status during deprivation process; PS-SD1, social interpersonal distance (1.2m) of peer social status during deprivation process; PS-PD1, personal interpersonal distance (0.6m) of peer social status during deprivation process; PS-ID1, intimate interpersonal distance (1.2m) of peer social status during deprivation process; DS-SD2, social interpersonal distance (1.2m) of differing social status during controllable process; DS-PD2, personal interpersonal distance (0.6m) of differing social status during controllable process; DS-ID2, intimate interpersonal distance (0.3m) of differing social status during controllable process; PS-SD2, social interpersonal distance (1.2m) of peer social status during controllable process; PS-PD2, personal interpersonal distance (0.6m) of peer social status during controllable process; PS-ID2, intimate interpersonal distance (0.3m) of peer social status during controllable process; t, t values; p, adjusted p values by the False Discovery Rate;

**Table 6: Activation channels in the IPD-Affection stage**

|  | **DS-POS-E** | | **DS-NEG-E** | | **DS-POS-A** | | **DS-NEG-A** | | **PS-POS-E** | | **PS-NEG-E** | | **PS-POS-A** | | **PS-NEG-A** | |
| --- | --- | --- | --- | --- | --- | --- | --- | --- | --- | --- | --- | --- | --- | --- | --- | --- |
| **Channels** | **t** | **p** | **t** | **p** | **t** | **p** | **t** | **p** | **t** | **p** | **t** | **p** | **t** | **p** | **t** | **p** |
| Channel 4 | 2.287 | 0.067 | 3.077 | 0.016 | 0.294 | 0.534 | -1.304 | 0.223 | 4.666 | 0.000 | 4.197 | 0.000 | -2.257 | 0.067 | -0.699 | 0.404 |
| Channel 5 | 1.709 | 0.148 | 1.965 | 0.107 | 0.397 | 0.510 | -0.516 | 0.467 | 3.400 | 0.010 | 3.352 | 0.009 | -0.135 | 0.561 | -1.759 | 0.145 |
| Channel 6 | 2.506 | 0.048 | 1.69 | 0.151 | -0.275 | 0.540 | -1.289 | 0.221 | 4.392 | 0.000 | 4.536 | 0.000 | -0.871 | 0.346 | -0.319 | 0.531 |
| Channel 7 | 4.192 | 0.000 | 3.233 | 0.010 | -0.894 | 0.344 | -1.752 | 0.145 | 3.606 | 0.007 | 4.525 | 0.000 | -0.983 | 0.314 | -1.563 | 0.174 |
| Channel 8 | 1.926 | 0.109 | 1.248 | 0.234 | 0.248 | 0.533 | -0.197 | 0.540 | 2.364 | 0.061 | 1.385 | 0.208 | 0.506 | 0.467 | -1.032 | 0.304 |
| Channel 9 | 1.043 | 0.308 | 0.342 | 0.534 | 0.729 | 0.398 | 0.215 | 0.540 | 2.367 | 0.059 | 2.501 | 0.051 | 0.586 | 0.449 | 0.089 | 0.565 |
| Channel 10 | 1.043 | 0.304 | 0.342 | 0.529 | 0.729 | 0.394 | 0.215 | 0.535 | 1.516 | 0.180 | 2.479 | 0.052 | 1.493 | 0.185 | 1.345 | 0.220 |
| Channel 11 | -1.321 | 0.220 | -1.734 | 0.147 | 1.294 | 0.224 | 0.693 | 0.403 | 2.353 | 0.057 | 1.536 | 0.180 | 1.007 | 0.311 | 0.559 | 0.458 |
| Channel 12 | 1.323 | 0.223 | 0.417 | 0.504 | 1.199 | 0.249 | -0.064 | 0.558 | 2.234 | 0.069 | 1.954 | 0.110 | 0.940 | 0.326 | -0.074 | 0.557 |
| Channel 13 | 1.093 | 0.290 | 0.638 | 0.427 | 1.398 | 0.210 | 0.952 | 0.324 | 1.987 | 0.108 | 1.948 | 0.107 | 1.524 | 0.181 | 0.038 | 0.565 |
| Channel 14 | -0.425 | 0.505 | -0.105 | 0.566 | 0.09 | 0.569 | -0.265 | 0.539 | 2.736 | 0.031 | 1.421 | 0.207 | 1.580 | 0.173 | 0.554 | 0.456 |
| Channel 15 | 1.992 | 0.107 | 1.646 | 0.157 | 0.137 | 0.565 | -0.832 | 0.357 | 3.004 | 0.017 | 3.783 | 0.006 | 0.295 | 0.539 | -0.081 | 0.559 |
| Channel 16 | 4.473 | 0.000 | 3.429 | 0.008 | 0.01 | 0.569 | -0.543 | 0.458 | 4.148 | 0.000 | 0.247 | 0.529 | -1.005 | 0.308 | -2.469 | 0.050 |
| Channel 17 | 0.088 | 0.560 | -0.25 | 0.542 | 0.878 | 0.348 | -0.136 | 0.556 | 4.388 | 0.000 | 3.487 | 0.009 | 0.248 | 0.538 | -1.920 | 0.109 |
| Channel 18 | 1.67 | 0.153 | 1.344 | 0.218 | 0.029 | 0.565 | -0.847 | 0.353 | 3.820 | 0.006 | 3.300 | 0.008 | -2.701 | 0.032 | -3.619 | 0.005 |
| Channel 19 | 2.026 | 0.105 | 2.031 | 0.106 | 0.323 | 0.534 | -0.787 | 0.374 | 5.073 | 0.000 | 3.212 | 0.011 | -1.410 | 0.207 | -1.709 | 0.150 |

DS-POS-E, evaluation periods after positive feedback in the differing social status condition; DS-NEG-E, evaluation periods after negative feedback in the differing social status condition; DS-POS-A, action periods after positive feedback in the differing social status condition; DS-NEG-A, action periods after negative feedback in the differing social status condition; PS-POS-E, evaluation periods after positive feedback in the peer social status condition; PS-NEG-E, evaluation periods after negative feedback in the peer social status condition; PS-POS-A, action periods after positive feedback in the peer social status condition; PS-NEG-A, action periods after negative feedback in the peer social status condition; t, t values; p, adjusted p values by the False Discovery Rate.

**Table 7: The Comparison of Oxy-Hb changes across channels of IPD-Inclusion stage (experiment 1)**

| **Channels** | **MNI**  **(x y z)** | **Activation**  **Brain regions** | **BA** | **Overlap**  **(%)** | **F** | **η²** | **PHMC/**  **Simple effect analysis** |
| --- | --- | --- | --- | --- | --- | --- | --- |
| **Social status (DS/PS)** |  |  |  |  |  |  |  |
| Channel 9 (n=37) | 27.4 69.1 5.6 | Frontopolar area  Orbitofrontal area | 10  11 | 72  28 | 7.365* | 0.170 | DS>PS |
| **IPDs (AIPD/PIPD)** |  |  |  |  |  |  |  |
| Channel 7 (n=37) | 38.3 59.5 16.5 | Frontopolar area  Dorsolateral prefrontal cortex | 10  46 | 46  54 | 4.166* | 0.104 | PIPD>AIPD |
| Channel 8 (n=31) | 26.9 59.7 29.9 | Dorsolateral prefrontal cortex  Frontopolar area  Dorsolateral prefrontal cortex | 9  10  46 | 20  30  50 | 6.951* | 0.188 | PIPD>AIPD |
| Channel 11 (n=37) | 4.3 63.6 30.4 | Dorsolateral prefrontal cortex  Frontopolar area | 9  10 | 18  82 | 4.494* | 0.111 | PIPD>AIPD |
| Channel 12 (n=37) | 3.8 71.6 5.9 | Frontopolar area | 10 | 100 | 5.485* | 0.132 | PIPD>AIPD |
| Channel 15 (n=36) | -22.1 69.9 4.1 | Frontopolar area  Orbitofrontal area | 10  11 | 68  32 | 7.873** | 0.184 | PIPD>AIPD |
| Channel 18 (n=37) | -43.8 55.7 -1 | Frontopolar area  Dorsolateral prefrontal cortex  Inferior prefrontal gyrus | 10  46  47 | -43.8 55.7 -1 | 8.621** | 0.193 | PIPD>AIPD |
| **Periods (evaluation/action)** |  |  |  |  |  |  |  |
| Channel 4 (n=35) | 55.4 38.4 11.8 | pars triangularis Broca's area  Dorsolateral prefrontal cortex | 45  46 | 97  3 | 10.755** | 0.240 | Action>Evaluation |
| Channel 6 (n=37) | 47.3 54.4 1.6 | Frontopolar area  Dorsolateral prefrontal cortex | 10  46 | 15  85 | 5.740* | 0.138 | Action>Evaluation |
| Channel 7 (n=37) | 38.3 59.5 16.5 | Frontopolar area  Dorsolateral prefrontal cortex | 10  46 | 46  54 | 5.434* | 0.131 | Action>Evaluation |
| Channel 11 (n=37) | 4.3 63.6 30.4 | Dorsolateral prefrontal cortex  Frontopolar area | 9  10 | 18  82 | 6.002* | 0.143 | Evaluation>Action |
| **Social status*IPDs** |  |  |  |  |  |  |  |
| Channel 4 (n=35) | 55.4 38.4 11.8 | pars triangularis Broca's area  Dorsolateral prefrontal cortex | 45  46 | 97  3 | 4.458* | 0.116 | PS, AIPD>PIPD |
| Channel 19 (n=33) | -52.4 40.6 8.5 | pars triangularis Broca's area  Dorsolateral prefrontal cortex | 45  46 | 85  15 | 8.545** | 0.211 | PIPD, DS>PS;  PS, AIPD>PIPD |
| **Social status*periods** |  |  |  |  |  |  |  |
| ns |  |  |  |  |  |  |  |
| **IPD*period** |  |  |  |  |  |  |  |
| ns |  |  |  |  |  |  |  |
|  |  |  |  |  |  |  |  |
| **Social status*IPDs*periods** |  |  |  |  |  |  |  |
| ns |  |  |  |  |  |  |  |

DS, differing social status; PS, peer social status; MNI, Montreal Neurological Institute; BA, Brodmann Area;

AIPD, active interpersonal distance; PIPD, passive interpersonal distance;

PHMC, post-hoc multiple comparison;

ns, no significant results; *, p < 0.05; **, p < 0.01. >, larger than: <, smaller than.

**Table 8: The Comparison of Oxy-Hb changes across channels in the IPD-Control stage (experiment 2)**

| **Channels** | **MNI**  **(x, y, z)** | **Activation brain regions** | **BA** | **Overlap**  **(%)** | **F** | **η²** | **PHMC / simple effects results** |
| --- | --- | --- | --- | --- | --- | --- | --- |
| **Social status (DS/PS)** |  |  |  |  |  |  |  |
| Channel 13 (n=36) | -11, 69.4, 18.1 | Frontopolar area | 10 | 100 | 4.863* | 0.122 | DS<PS |
| **Processes**  (deprivation/controllable) |  |  |  |  |  |  |  |
| Channel 4 (n=35) | 55.4 38.4 11.8 | pars triangularis Broca's area  Dorsolateral prefrontal cortex | 45  46 | 97  3 | 28.596*** | 0.457 | deprivation>controllable |
| Channel 6 (n=37) | 47.3, 54.4, 1.6 | Frontopolar area  Dorsolateral prefrontal cortex | 10  46 | 15  85 | 9.357** | 0.206 | deprivation>controllable |
| Channel 17 (n=37) | -44.1, 47.2, 24.4 | pars triangularis Broca’s area  Dorsolateral prefrontal cortex | 45  46 | 55  45 | 6.134* | 0.146 | deprivation>controllable |
| Channel 19 (n=34) | -52.4, 40.6, 8.5 | pars triangularis Broca’s area  Dorsolateral prefrontal cortex | 45  46 | 85  15 | 17.432*** | 0.346 | deprivation>controllable |
| **IPDs**  **[social IPD (1.2m)/personal IPD (0.6m)/intimate IPD (0.3m)]** |  |  |  |  |  |  |  |
| Channel 5 (n=37) | 47.4, 44.6, 26.4 | pars triangularis Broca’s area  Dorsolateral prefrontal cortex | 45  46 | 72  28 | 6.651** | 0.156 | Intimate IPD (0.3m)>social IPD (1.2m), intimate IPD (0.3m)>personal IPD (0.6m) |
| Channel 7 (n=37) | 38.3, 59.5, 16.5 | Frontopolar area  Dorsolateral prefrontal cortex | 10  46 | 46  54 | 3.270* | 0.083 | Intimate IPD (0.3m)>social IPD (1.2m) |
| Channel 8 (n=33) | 26.9 59.7 29.9 | Dorsolateral prefrontal cortex  Frontopolar area  Dorsolateral prefrontal cortex | 9  10  46 | 20  30  50 | 3.780* | 0.106 | Intimate IPD (0.3m)>personal IPD (0.6m) |
| Channel 9 (n=37) | 27.4 69.1 5.6 | Frontopolar area  Orbitofrontal area | 10  11 | 72  28 | 4.020* | 0.100 | Intimate IPD (0.3m)>personal IPD (0.6m) |
| Channel 10 (n=36) | 16.9, 69.1, 19 | Frontopolar area | 10 | 100 | 6.017** | 0.147 | Intimate IPD (0.3m)>social IPD (1.2m), intimate IPD (0.3m)>personal IPD (0.6m) |
| Channel 11 (n=36) | 4.3, 63.6, 30.4 | Dorsolateral prefrontal cortex  Frontopolar area | 9  10 | 18  82 | 9.794*** | 0.214 | Intimate IPD (0.3m)>social IPD (1.2m), intimate IPD (0.3m)>personal IPD (0.6m) |
| Channel 12 (n=37) | 3.8, 71.6, 5.9 | Frontopolar area | 10 | 100 | 5.020** | 0.122 | Intimate IPD (0.3m)>social IPD (1.2m); |
| Channel 13 (n=36) | -11, 69.4, 18.1 | Frontopolar area | 10 | 100 | 4.984** | 0.125 | Intimate IPD (0.3m)>social IPD (1.2m), intimate IPD (0.3m)>personal IPD (0.6m); |
| Channel 14 (n=35) | -21.5 61.6 29 | Dorsolateral prefrontal cortex  Frontopolar area  Dorsolateral prefrontal cortex | 9  10  46 | 18  48  34 | 7.433** | 0.179 | Intimate IPD (0.3m)>social IPD (1.2m), intimate IPD (0.3m)>personal IPD (0.6m); |
| Channel 15 (n=36) | -22.1 69.9 4.1 | Frontopolar area  Orbitofrontal area | 10  11 | 68  32 | 3.856* | 0.099 | - |
| **Social status* Processes** |  |  |  |  |  |  |  |
| Channel 10 (n=36) | 16.9, 69.1, 19 | Frontopolar area | 10 | 100 | 6.316* | 0.153 | Deprivation, DS>PS |
| Channel 12 (n=37) | 3.8, 71.6, 5.9 | Frontopolar area | 10 | 100 | 5.836* | 0.139 | Deprivation, DS>PS |
| Channel 13 (n=36) | -11, 69.4, 18.1 | Frontopolar area | 10 | 100 | 8.114** | 0.188 | Controllable, DS<PS; |
| Channel 14 (n=35) | -21.5 61.6 29 | Dorsolateral prefrontal cortex  Frontopolar area  Dorsolateral prefrontal cortex | 9  10  46 | 18  48  34 | 6.939* | 0.169 | Controllable, DS<PS; |
| Channel 15 (n=36) | -22.1 69.9 4.1 | Frontopolar area  Orbitofrontal area | 10  11 | 68  32 | 5.884* | 0.144 | - |
| Channel 16 (n=36) | -34.6, 61.4, 14.4 | Frontopolar area  Dorsolateral prefrontal cortex | 10  46 | 59  41 | 12.880** | 0.269 | Controllable, DS<PS; |
| Channel 17 (n=37) | -44.1, 47.2, 24.4 | pars triangularis Broca’s area  Dorsolateral prefrontal cortex | 45  46 | 55  45 | 5.865* | 0.140 | Controllable, DS<PS;  DS, deprivation>controllable |
| Channel 18 (n=37) | -43.8,55.7, -1 | Frontopolar area  Dorsolateral prefrontal cortex  Inferior prefrontal gyrus | 10  46  47 | 24  75  1 | 5.182* | 0.126 | Controllable, DS<PS;  DS, deprivation>controllable |
| **Social status*IPDs** |  |  |  |  |  |  |  |
| ns |  |  |  |  |  |  |  |
| **Processes*IPDs** |  |  |  |  |  |  |  |
| Channel 4 (n=35) | 55.4, 38.4, 11.8 | pars triangularis Broca’s area  Dorsolateral prefrontal cortex | 45  46 | 97  3 | 3.549* | 0.095 | IPDs, deprivation>controllable |
| Channel 5 (n=37) | 47.4, 44.6, 26.4 | pars triangularis Broca’s area  Dorsolateral prefrontal cortex | 45  46 | 72  28 | 8.034** | 0.182 | Controllable, Intimate IPD (0.3m)> Social IPD (1.2m); intimate IPD (0.3m)>personal IPD (0.6m) |
| Channel 7 (n=37) | 38.3, 59.5, 16.5 | Frontopolar area  Dorsolateral prefrontal cortex | 10  46 | 46  54 | 6.383** | 0.151 | Controllable, Intimate IPD (0.3m)> Social IPD (1.2m); intimate IPD (0.3m)>personal IPD (0.6m); |
| Channel 10 (n=36) | 16.9, 69.1, 19 | Frontopolar area | 10 | 100 | 3.934* | 0.101 | Controllable, Intimate IPD (0.3m)> Social IPD (1.2m); intimate IPD (0.3m)>personal IPD (0.6m); |
| **Social status* Processes *IPDs** |  |  |  |  |  |  |  |
| Channel 7 (n=37) | 38.3, 59.5, 16.5 | Frontopolar area  Dorsolateral prefrontal cortex | 10  46 | 46  54 | 3.752* | 0.094 | DS, Social IPD (1.2m), deprivation>controllable;  DS, controllable, Intimate IPD (0.3m)> Social IPD (1.2m);  PS, controllable, Intimate IPD (0.3m)> personal IPD (0.6m); |
| Channel 9 (n=37) | 27.4 69.1 5.6 | Frontopolar area  Orbitofrontal area | 10  11 | 72  28 | 5.318** | 0.129 | Deprivation, DS>PS;  PS, Intimate IPD (0.3m), DS<PS;  PS, controllable, Intimate IPD (0.3m)> personal IPD (0.6m); |
| Channel 10 (n=36) | 16.9, 69.1, 19 | Frontopolar area | 10 | 100 | 3.593* | 0.093 | Deprivation, Intimate IPD (0.3m), DS>PS;  Controllable, Social IPD (1.2m)/ Intimate IPD (0.3m) DS<PS; |
| Channel 12 (n=37) | 3.8, 71.6, 5.9 | Frontopolar area | 10 | 100 | 5.674** | 0.136 | Deprivation, Intimate IPD (0.3m), DS>PS;  Controllable, social IPD (1.2m), PS>DS;  Controllable, intimate IPD (0.3m), PS>DS; |

DS, differing social status; PS, peer social status; MNI, Montreal Neurological Institute; BA, Brodmann Area; PHMC, post-hoc multiple comparison;

ns, no significant results; *, p < 0.05; **, p < 0.01; ***, p < 0.001. >, larger than: <, smaller than.

Social IPD (1.2m)/personal IPD (0.6m)/intimate IPD (0.3m), during the action period of social IPD (1.2m)/personal IPD (0.6m)/intimate IPD (0.3m). intimate IPD (0.3m)>personal IPD (0.6m), Oxy-Hb changes in the intimate IPD (0.3m) were larger than personal IPD (0.6 m) during the action period. intimate IPD (0.3m)> social IPD (1.2m), Oxy-Hb changes in the intimate IPD (0.3m) were larger than social IPD (1.2m) during the action period. personal IPD (0.6 m)> social IPD (1.2m), Oxy-Hb changes in the personal IPD (0.6 m) were larger than social IPD (1.2m) during the action period.

**Table 9: The comparison of [Oxy-Hb] of each channel in the IPD-Affection stage (experiment 3)**

| **Channels** | **MNI**  **(x y z)** | **Activation**  **Brain regions** | **BA** | **Overlap**  **(%)** | **F** | **η²** | **PHMC/**  **Simple effect analysis** |
| --- | --- | --- | --- | --- | --- | --- | --- |
| **Social status (DS/PS)** |  |  |  |  |  |  |  |
| Channel 17 (n=24) | -44.1 47.2 24.4 | pars triangularis Broca's area  Dorsolateral prefrontal cortex | 45  46 | 55  45 | 4.728* | 0.171 | DS<PS |
| **IPD feedback (POS/NEG)** |  |  |  |  |  |  |  |
| Channel 8 (n=21) | 26.9 59.7 29.9 | Dorsolateral prefrontal cortex  Frontopolar area  Dorsolateral prefrontal cortex | 9  10  46 | 20  30  50 | 11.774** | 0.371 | POS>NEG |
| Channel 9 (n=24) | 27.4 69.1 5.6 | Frontopolar area  Orbitofrontal area | 10  11 | 72  28 | 4.863* | 0.175 | POS>NEG |
| Channel 10 (n=24) | 16.9 69.1 19 | Frontopolar area | 10 | 100 | 8.619** | 0.273 | POS>NEG |
| Channel 11 (n=24) | 4.3 63.6 30.4 | Dorsolateral prefrontal cortex  Frontopolar area | 9  10 | 18  82 | 7.665* | 0.250 | POS>NEG |
| Channel 12 (n=24) | 3.8 71.6 5.9 | Frontopolar area | 10 | 100 | 12.031** | 0.343 | POS>NEG |
| Channel 16 (n=23) | -34.6 61.4 14.4 | Frontopolar area  Dorsolateral prefrontal cortex | 10  46 | 59  41 | 6.600* | 0.231 | POS>NEG |
| **Periods (evaluation/action)** |  |  |  |  |  |  |  |
| Channel 4 (n=23) | 55.4 38.4 11.8 | pars triangularis Broca's area  Dorsolateral prefrontal cortex | 45  46 | 97  3 | 13.496** | 0.380 | Evaluation>Action |
| Channel 5 (n=24) | 47.4 44.6 26.4 | pars triangularis Broca's area  Dorsolateral prefrontal cortex | 45  46 | 72  28 | 4.530* | 0.165 | Evaluation>Action |
| Channel 6 (n=24) | 47.3 54.4 1.6 | Frontopolar area  Dorsolateral prefrontal cortex | 10  46 | 15  85 | 13.777** | 0.375 | Evaluation> Action |
| Channel 7 (n=24) | 38.3 59.5 16.5 | Frontopolar area  Dorsolateral prefrontal cortex | 10  46 | 46  54 | 20.017*** | 0.465 | Evaluation> Action |
| Channel 16 (n=23) | -34.6 61.4 14.4 | Frontopolar area  Dorsolateral prefrontal cortex | 10  46 | 59  41 | 11.080** | 0.335 | Evaluation> Action |
| Channel 18 (n=24) | -43.8 55.7 -1 | Frontopolar area  Dorsolateral prefrontal cortex  Inferior prefrontal gyrus | 10  46  47 | 24  75  1 | 8.456** | 0.269 | Evaluation> Action |
| Channel 19 (n=22) | -52.4 40.6 8.5 | pars triangularis Broca's area  Dorsolateral prefrontal cortex | 45  46 | 85  15 | 9.702** | 0.316 | Evaluation> Action |
| **Social status *IPD Feedback** |  |  |  |  |  |  |  |
| Channel 14 (n=24) | -21.5 61.6 29 | Dorsolateral prefrontal cortex  Frontopolar area  Dorsolateral prefrontal cortex | 9  10  46 | 18  48  34 | 6.031* | 0.208 | PS, POS>NEG |
| Channel 16 (n=23) | -34.6 61.4 14.4 | Frontopolar area  Dorsolateral prefrontal cortex | 10  46 | 59  41 | 6.314* | 0.223 | POS, DS<PS;  PS, POS >NEG |
| **Social status*periods** |  |  |  |  |  |  |  |
| Channel 19 (n=22) | -52.4 40.6 8.5 | pars triangularis Broca's area  Dorsolateral prefrontal cortex | 45  46 | 85  15 | 4.507* | 0.177 | Action, DS > PS;  PS, evaluation >action |
| **IPD feedback * period** |  |  |  |  |  |  |  |
| Channel 13 (n=23) | -11 69.4 18.1 | Frontopolar area | 10 | 100 | 7.176* | 0.246 | Action, POS>NEG |
| Channel 17 (n=24) | -44.1 47.2 24.4 | pars triangularis Broca's area  Dorsolateral prefrontal cortex | 45  46 | 55  45 | 4.433* | 0.162 | NEG, Evaluation > Action |
| **Social status *IPD feedback* periods** |  |  |  |  |  |  |  |
| ns |  |  |  |  |  |  |  |

MNI, Montreal Neurological Institute; BA, Brodmann Area; DS, differing social status; PS, peer social status; POS, positive interpersonal distance feedback; NEG, negative interpersonal distance feedback; ns, none significant results, *, p < 0.05; **, p < 0.01; ***, p < 0.001. >, larger than: <, smaller than.
