# Supplementary figures and images for: Neural and behavioral alterations of a real-time interpersonal distance (IPD) development process in differing social status interactions
Source: Front Behav Neurosci. 2022 Oct 13;16:969440. doi: 10.3389/fnbeh.2022.969440 (PMC9616044; doi:10.3389/fnbeh.2022.969440)

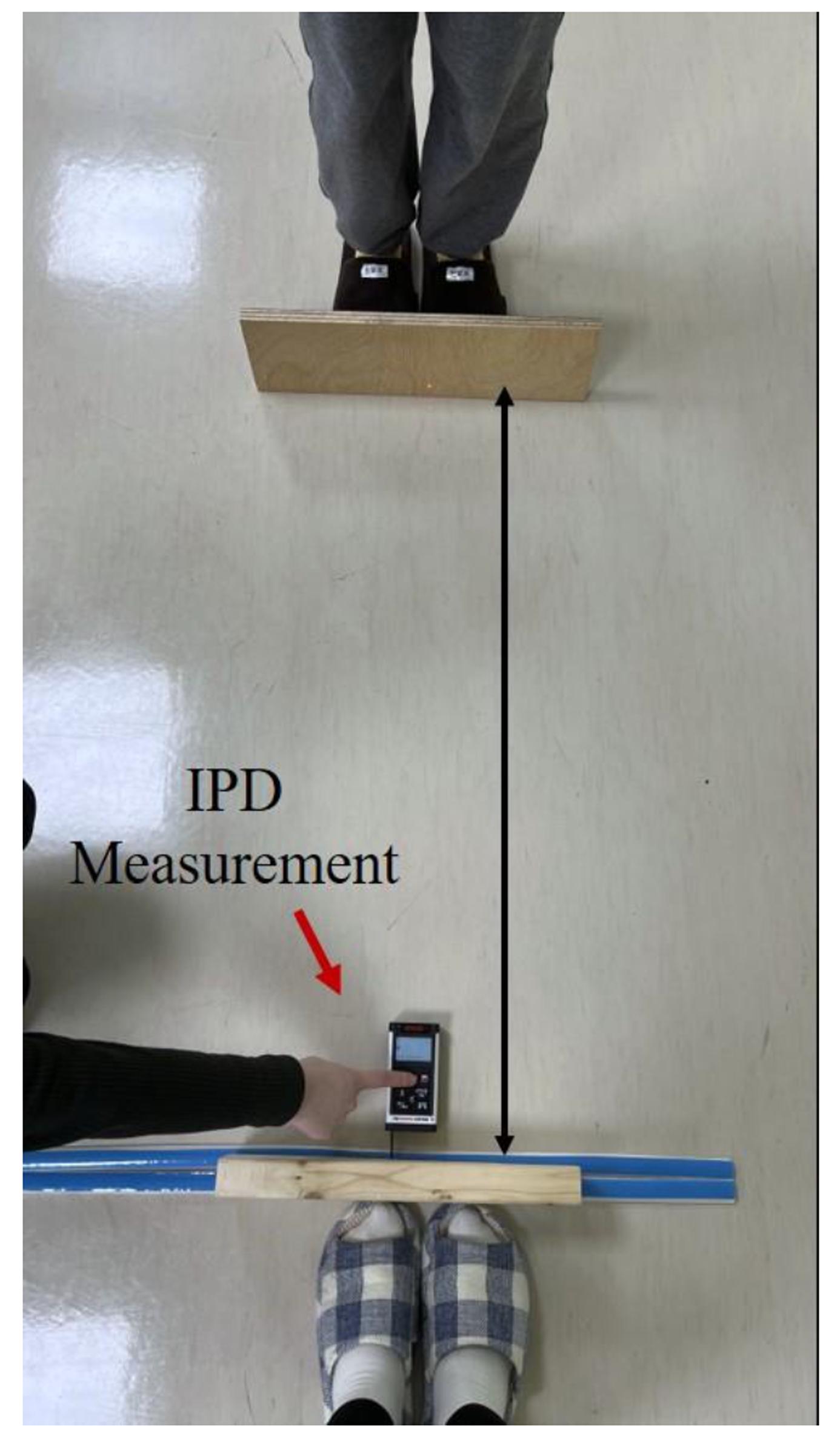

Supplement: Supplementary file 2 [file Image_1.JPEG]

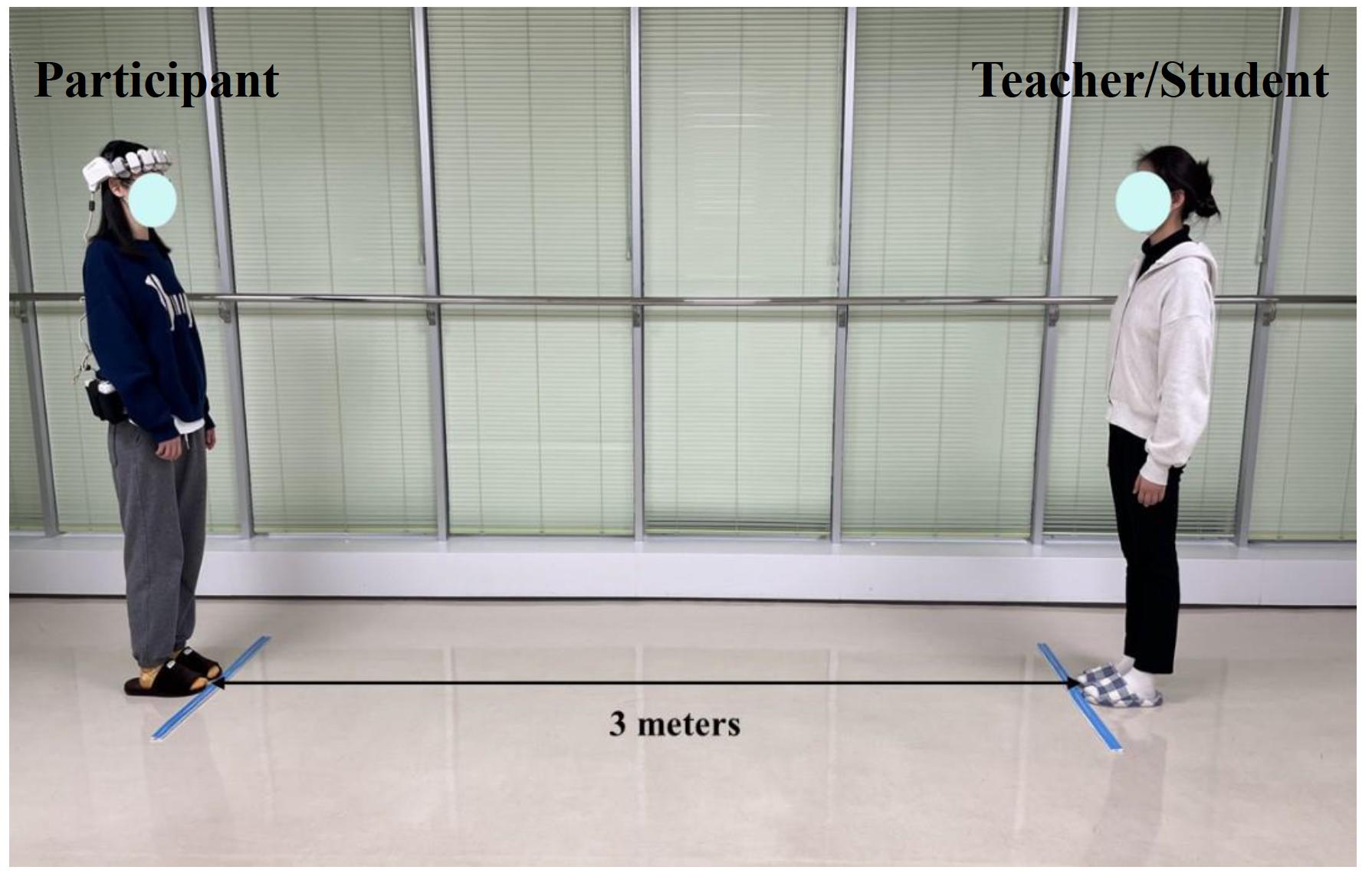

Supplement: Supplementary file 3 [file Image_2.JPEG]
